# Consequences of benzalkonium chloride tolerance for selection dynamics and de novo resistance evolution driven by antibiotics

**DOI:** 10.1038/s44259-025-00170-8

**Published:** 2026-01-08

**Authors:** Orestis Kanaris, Lydia-Yasmin Sobisch, Annett Gödt, Frank Schreiber, Niclas Nordholt

**Affiliations:** Division of Biodeterioration and Reference Organisms (4.1), Department of Materials and the Environment, Federal Institute for Materials Research and Testing (BAM), Berlin, Germany

**Keywords:** Microbiology, Molecular biology

## Abstract

Biocides are used in large amounts in industrial, medical, and domestic settings. Benzalkonium chloride (BAC) is a commonly used biocide, for which previous research revealed that *Escherichia coli* can rapidly adapt to tolerate BAC-disinfection, with consequences for antibiotic susceptibility. However, the consequences of BAC tolerance for selection dynamics and resistance evolution to antibiotics remain unknown. Here, we investigated the effect of BAC tolerance in *E. coli* on its response upon challenge with different antibiotics. Competition assays showed that subinhibitory concentrations of ciprofloxacin—but not ampicillin, colistin and gentamicin—select for the BAC-tolerant strain over the BAC-sensitive ancestor at a minimal selective concentration of 0.0013–0.0022 µg∙mL^−^^1^. In contrast, the BAC-sensitive ancestor was more likely to evolve resistance to ciprofloxacin, colistin and gentamicin than the BAC-tolerant strain when adapted to higher concentrations of antibiotics in a serial transfer laboratory evolution experiment. The observed difference in the evolvability of resistance to ciprofloxacin was partly explained by an epistatic interaction between the mutations conferring BAC tolerance and a knockout mutation in *ompF* encoding for the outer membrane porin F. Taken together, these findings suggest that BAC tolerance can be stabilized in environments containing low concentrations of ciprofloxacin, while it also constrains evolutionary pathways towards antibiotic resistance.

## Introduction

The widespread use of antiseptics and biocides has raised concerns regarding the emergence and spread of multi-drug resistance^1^. However, there is limited understanding of the effect of antiseptics and biocides on the selection and development of antibiotic resistance^2^. Antiseptics and biocides are products that eliminate or inhibit harmful microorganisms. Both types of products may contain similar active substances, but they are used in different applications and are thus regulated by different laws in the EU^3,4^. Antiseptics are applied to living tissues, such as wounds or surgical sites, to prevent or treat infections^4^. In contrast, biocides are used in various environments, including industry, hospitals, agriculture, and household products, to control harmful organisms, such as disinfectants for inanimate surfaces and preservatives^1^. While antiseptics and biocides are in many cases based on active substances with a broad antimicrobial effect, active substances are used in antibiotics as pharmaceuticals for the systemic treatment of humans or animals mostly relying on defined biochemical targets in bacteria.

Biocides used as disinfectants play a crucial role in medical and veterinary settings, especially since the worldwide rise of antibiotic resistance^5,6^. Ever since the COVID-19 pandemic the use of biocides is increasing^7^. Concerns have been raised that biocide exposure may select for antibiotic resistance, particularly after repeated exposure to biocides at subinhibitory concentrations such as BAC or triclosan^8,9^. Several overlapping molecular resistance mechanisms have been identified. For example, efflux pumps such as AcrAB-TolC and the activity of transcriptional regulators like MarA can lead to reduced intracellular accumulation of both biocides, such as quaternary ammonium compounds and phenolics, and antibiotics, such as tetracyclines and fluoroquinolones^10,11^. Similarly, changes in outer membrane permeability or modifications in lipopolysaccharides (LPS) can confer decreased susceptibility to both substance classes^2,12,13^. These overlapping mechanisms demonstrate how adaptation to biocides may generate cross-resistance to other biocides and clinically relevant antibiotics^1,14^. This highlights the need to investigate the consequences of the widespread use of biocides on resistance to antibiotics and biocides in order to identify risks of biocide use and to inform regulatory interventions.

Microorganisms are categorized as resistant to a given antibiotic when they possess an inherited ability which allows them to grow at antibiotic concentrations that otherwise inhibit the growth of related strains^15^. Clinically, resistance to an antibiotic is scored if this difference in susceptibility leads to a high probability of treatment failure^15^. Resistance can be determined by measuring the minimal inhibitory concentration (MIC), the lowest antibiotic concentration that prevents visible growth. In contrast, tolerance refers to the ability of a microbial strain to survive exposure to an antimicrobial substance (e.g. antibiotics or biocides) for longer durations than a related, susceptible strain. In many cases, tolerance is not associated to increases in MIC and resistance^15,16^. Tolerance is the main mode of microorganisms to evade the effects of biocides used as disinfectants because their efficacy is based upon killing. Tolerant bacteria can slow or pause their growth in the presence of antimicrobials that require growth to harm the bacteria. While resistance and tolerance are often discussed together, they rely on distinct molecular mechanisms^16^, a point underscored by the observation that tolerance can increase without changes in MIC. For example, Zeng and colleagues^17^ demonstrated that repeated low-level biocide exposure selects for bacterial populations with complex survival strategies that may cross-protect against structurally and functionally different stressors, indicating potential links between biocide tolerance and multi-drug resistance. Moreover, it has been shown that antibiotic tolerance can facilitate the evolution of resistance to the same antibiotic^18^. Our previous work has shown that high levels of disinfectant tolerance evolve under periodic exposure^19,20^. These biocide-tolerant strains possess unique survival mechanisms that could have two consequences upon challenge with antibiotics; first, the decreased susceptibility may facilitate their selection in competition with sensitive strains and second, the differences in genetic make-up might impact their subsequent evolution of higher levels of resistance to biocides as well as antibiotics^21,22^. However, the response of biocide-tolerant strains upon challenge with antibiotics has not been studied in detail.

Such above discussed consequences might be especially relevant in environments, in which biocides and antibiotics co-occur along with resistant or tolerant strains. Co-occurrence of antimicrobial substances like biocides and antibiotics happens in areas such as hospitals, animal husbandry, veterinary settings, and wastewater treatment^1,22,23^. It has been previously reported that bacteria that were in contact with the biocide BAC, developed a slightly reduced susceptibility to the antibiotics ampicillin (AMP) and ciprofloxacin (CIP) than before the exposure to BAC^9,19,24^. In addition, BAC-tolerant strains exhibited a higher growth rate as compared to their sensitive ancestral strain under subinhibitory concentrations of CIP^19^. Whether these slight fitness benefits alter the ability of the biocide-tolerant strains to be selected in mixed populations or if they facilitate the evolution of higher levels of antibiotic resistance is unknown.

Selection describes a dynamic process within populations of organisms in which the proportion of a specific genotype increases as compared to another genotype because the increasing genotype is favored by the environmental conditions (i.e. it has higher fitness). The presence of subinhibitory antibiotic concentrations can lead to fitness differences between genotypes and thus can drive selection of specific genotypes^25^. Increased fitness of resistant or tolerant strains over susceptible ones can lead to the selection, and subsequently to the dominance of the resistant or tolerant strains and the spread of antimicrobial resistance or tolerance in mixed populations. Under sub-MIC concentrations of antibiotics, the selection of resistant bacterial strains can be favored in direct competition over susceptible ones even at very low antibiotic concentrations (>230 times below the MIC). The lowest concentration of an antibiotic capable of selecting for the resistant genotype is defined as the minimal selective concentration (MSC)^26^. A strain with decreased susceptibility to a biocide (either by increased growth or increased survival) may display cross-resistance to antibiotics. Such cross-resistance can cause selection of this strain in the presence of antibiotics; this phenomenon is termed co-selection^23^. Currently, it is unknown whether biocide tolerance, which might be associated with fitness costs, provides a fitness advantage and thus allows co-selection in the presence of antibiotics, and thereby is stabilized in the presence of antibiotics at environmentally relevant concentrations.

Biocide-tolerant strains may not only be affected by antibiotics through differences in their competitive ability and thus co-selection. In addition, biocide-tolerant strains may differ in their ability to evolve in the face of a challenge with high levels of antibiotics. The evolvability of resistance describes the ability of a microorganism to develop resistance to an antimicrobial agent and thereby maintain growth under inhibitory antimicrobial concentrations^27,28^. The evolvability of antibiotic resistance in biocide-tolerant strains can be affected by changes in the mutation rate (i.e. the rate at which mutations occur) and epistatic interactions between genes or mutations that confer biocide tolerance with those genes or mutations that confer antibiotic resistance^29,30^. However, it is unknown whether tolerance to biocides affects the evolvability of antibiotic resistance. Determining the effects of evolved biocide tolerance on evolvability of antibiotic resistance is important for assessing the risks of biocide usage and subsequent emergence and fate of biocide-tolerant strains, especially if those strains can cause infections that need to be treated with antibiotics.

In this study, we experimentally investigate the effect of previously evolved BAC tolerance on the trajectory of two distinct evolutionary processes: (i) the selection dynamics of a BAC-tolerant strain in competition with its susceptible ancestor in the presence of subinhibitory antibiotic concentrations and (ii) the de novo evolution of antibiotic resistance in a BAC-tolerant strain as compared to its susceptible ancestor at inhibitory antibiotic concentrations. Both processes are important to understand the fate of BAC-tolerant strains either in (i) environments which are contaminated with low levels of antibiotics, e.g. wastewater, or (ii) in environments with clinically relevant concentrations of antibiotics, e.g. infected patients during antibiotic treatment. Selection dynamics were investigated through competition experiments, while differences in the ability to evolve antibiotic resistance were assessed through parallel serial transfer evolution experiments. The experiments were performed with antibiotics from different classes (AMP: beta-lactams, CIP: fluoroquinolones, colistin, COL: polymyxin and gentamycin, GEN: aminoglycosides) to uncover potential generalizable mechanisms across antibiotics. Both evolutionary processes were assessed by focusing on the highly BAC-tolerant strain S4, which was previously isolated from a laboratory evolution experiment under periodic exposure to BAC and comparing it with its ancestor strain^19^. The BAC-tolerant strain was selected because we previously found that it exhibited the highest increase in growth rate as compared to the wildtype (WT) strain in the presence of subinhibitory concentrations of CIP, along with a fitness cost (19 % compared to the ancestor) related to BAC tolerance^19^. We find that BAC tolerance impacts selection in the presence of antibiotics and evolvability of antibiotic resistance. Specifically, CIP leads to selection of the BAC-tolerant strain S4, while the same strain showed decreased evolvability of CIP resistance. The decrease in evolvability of CIP resistance was confirmed to occur in other laboratory-evolved BAC-tolerant strains. We then performed focused mechanistic experiments with strain S4 and CIP, revealing the outer membrane porin F (*ompF*) is a genetic factor that underpins the decreased evolvability using whole genome sequencing and targeted mutagenesis.

## Results and discussion

### Subinhibitory concentrations of CIP select for BAC tolerance

**Fig. 1:**
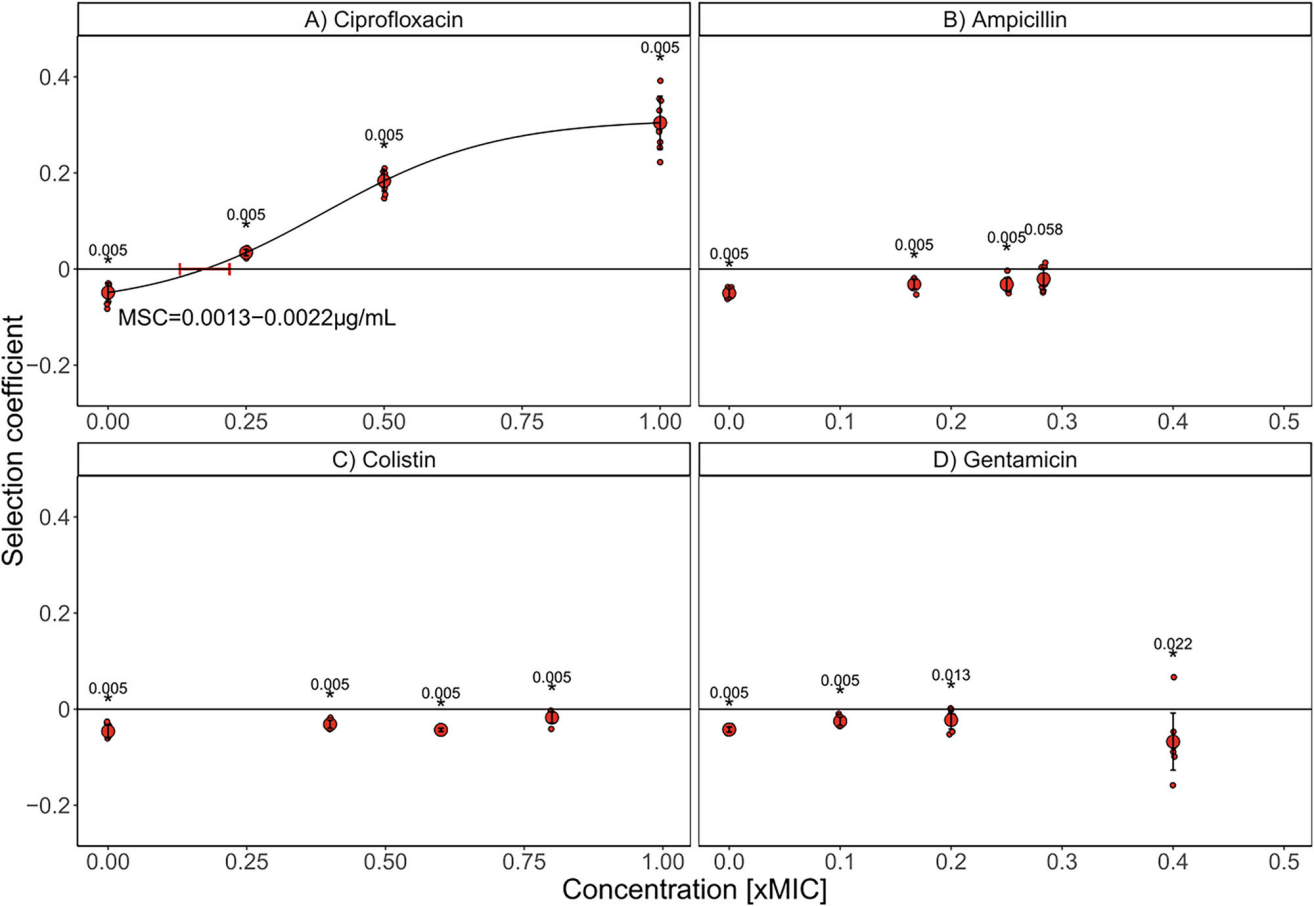
Effect of antibiotics on the selection of the benzalkonium chloride (BAC)-tolerant strain S4 in competition with its ancestor (*E. coli* MG1655 wildtype, WT). Competition experiments were performed in the presence of (**A**) ciprofloxacin (CIP), (**B**) ampicillin (AMP), (**C**) colistin (COL), and (**D**) gentamicin (GEN). Competitions between the WT tagged with mCherry and the BAC-tolerant strain S4 tagged with YFP were performed with 9 replicate lines under three different antibiotic concentrations (CIP: 0; 0.0025; 0.005; 0.01 µg∙mL^−1^, AMP: 0; 0.5; 0.75; 0.85 µg∙mL^−1^, COL: 0; 0.2; 0.3; 0.4 µg∙mL^−1^, GEN: 0; 0.05; 0.1; 0.2 µg∙mL^−1^). The panels display the selection coefficient of S4 against the WT as a function of the concentration represented as the fold-difference to the minimum inhibitory concentration (MIC) of the WT (CIP: 0.01 µg∙mL^−1^; AMP: 4 µg∙mL^−1^; COL: 0.5 µg∙mL^−1^; GEN: 0.5 µg∙mL^−1^). The selection coefficient was calculated from the change of S4 relative to the WT over generations of competition (as calculated from data shown in Supplementary Fig. S3). The regression of the experimental results (black line) in A was fitted with a logistic function including all nine competitions per concentration (small circles). The intercept with the x-axis represents the minimum selection concentration (MSC). Positive values on the y-axis indicate selection for S4 and negative values selection for the WT. The red, big circles show the mean of the individual replicates. The error bars show the standard deviation. Outcomes of competitions with reciprocal fluorescent tags are shown in Supplementary Figs. S1 and S4. One-sample Wilcoxon test was performed on the data of the 9 replicates to test for a significant deviation from 0 and the *p*-values are shown above each concentration (stars indicate significance at *p* < 0.05 corrected for multiple comparisons with the method from Benjamini & Hochberg^41^).

Competition experiments were conducted to understand the selection dynamics between the BAC-tolerant strain S4 and its ancestor *E. coli* strain (WT) under the influence of antibiotics chosen to represent widely different antibiotic classes (AMP: beta-lactams, CIP: fluoroquinolones, COL: polymyxin and GEN: aminoglycosides). Each of the two strains were fluorescently tagged with the fluorescent proteins YFP and mCherry and analyzed in competition experiments using various subinhibitory concentrations of the antibiotics. Competition experiments proceeded over ~20 generations because fixation of resistant mutants is not expected within this timeframe^25^. The experiments were carried out for both fluorescence combinations; WT expressing YFP competing with S4 expressing mCherry (Fig. 1) and WT expressing mCherry competing with S4 expressing YFP (Supplementary Fig. S1) to control for effects caused by the fluorescent proteins. The data showed that the choice of fluorescent proteins had a significant effect for 2 out of 16 specific combinations of antibiotics and their concentrations (Supplementary Fig. S2, Scheirer-Ray-Hare test followed by Dunn’s posthoc test at *p* < 0.05 for each antibiotic with factors fluorescent protein and antibiotic concentration). For this reason, the two combinations were analyzed separately and only those effects that were significant in the same direction of the effect in both fluorescence combinations were considered as true effects.

The data showed that the BAC-tolerant strain S4 exhibits a fitness advantage in the presence of increasing CIP concentrations, outcompeting the WT for both fluorescence combinations (Fig. 1A and Supplementary Fig. S1A). The MSC of CIP for the BAC-tolerant S4 strain was determined to range between 0.0013 and 0.0022 µg∙mL^−1^ (Fig. 1) and 0.0017–0.0023 µg∙mL^−1^ (Supplementary Fig. S1), demonstrating that the fluorescent protein had only a minor effect on the estimation of the MSC. The estimated range of the MSC is ca. 4-fold lower than MIC (MIC_WT_ = 0.01 µg∙mL^−1^). The competitive advantage of the BAC-tolerant strain S4 can be explained by its higher growth rate as compared to the WT in the presence of CIP^19^. In contrast, AMP, COL, and GEN did not select for the BAC-tolerant strain (Fig. 1B-D). This suggests that BAC tolerance does not generally convey a selective advantage in the presence of antibiotics. Rather, the selective advantage depends on the molecular mechanism of BAC tolerance and how it modulates the efficacy of specific antibiotics. Even though GEN did not select for the tolerant strain consistently across both fluorescent combinations, the highest GEN concentration tested led to very diverse outcomes between the replicates (Fig. 1D and Supplementary Fig. 1D). This indicates that subinhibitory concentrations of GEN close to the MIC can disturb the balance between different genotypes and lead to unpredictable population dynamics. Taken together, the results of the competition experiments demonstrate that subinhibitory concentrations of ciprofloxacin can select for a biocide-tolerant strain in direct competition with a sensitive strain, but such positive selection is not a generalizable trend across other classes of antibiotics.

The mutations in the BAC-tolerant strain S4 are not associated to high CIP resistance^19^. This strain carries a mutation in *lpxM*, which catalyzes the last step of the lipid A biosynthesis and a loss-of-function mutation in *rssB* (Table 1). RssB regulates the degradation of RpoS, the master regulator of the general stress response^31,32^. Non-functioning RssB leads to accumulation of RpoS in the cell, which results in the sustained activation of the general stress response^32^, which might be related to the strong increase in BAC tolerance and the associated modest increase in CIP resistance. However, this hypothesis has not been tested experimentally. Nevertheless, in direct competition with the susceptible WT, the BAC-tolerant strain exhibited a similar MSC to that of *E. coli* strains carrying clinically relevant CIP resistance mutations (e.g., *gyrA* D87N, Δa*crR*, and Δ*marR*). In previous studies, such resistance mutations were shown to have MSCs of approximately 0.1-fold of the MIC of the susceptible parental strain^25^. This suggests that the BAC-tolerant strain, despite not being resistant in terms of MIC, possesses a relevant selective advantage under subinhibitory CIP concentrations against the WT. The increased growth rate of S4 in presence of subinhibitory CIP^19^ likely underlies the selective advantage over the ancestor. However, the molecular basis for the growth advantage remains unknown. In Germany, CIP concentrations of similar range as the determined MSC (0.0013–0.022 µg∙mL^−1^) were found in wastewater samples^33^. Specifically, in hospital wastewaters and sewage sludge, maximum concentrations of ~0.06 µg∙mL^-−1^ and 0.6 µg∙mL^−1^ were reported, respectively, corresponding to ~30-fold and 300-fold higher than the MSC of the BAC-tolerant strain determined here. In addition, in pig and poultry slurry in Germany in 2018, a maximum of 0.1 µg∙g^−1^ CIP was recorded, i.e. approximately 50-fold higher than the determined MSC in this present study^33^. Considering that BAC is widely used as a disinfectant and cleaning agent in medical and animal husbandry, there is a risk that strains like the BAC-tolerant strain S4 could evolve in these environments. The concomitant presence of subinhibitory levels of CIP could alleviate the fitness costs of BAC tolerance and contribute to the selection of BAC-tolerant strains. As a possible consequence, disinfectant-tolerant bacteria could persist in these settings and constantly be released into the environment through the waste. Colonization with such bacteria could render BAC applied as disinfectant or antiseptic less effective.

**Table 1:** Strains used in this study including their genotype and their minimum inhibitory concentrations

Designation in main text *E. coli* strains	Genotype	Minimum inhibitory concentration				Origin of Strain, Reference
		AMP (µg ∙ mL^−1^)	CIP (µg ∙ mL^−1^)	COL (µg ∙ mL^−1^)	GEN (µg ∙ mL^−1^)	
MG1655 (wildtype, WT or ancestor)	Δ776 bp in *crl*, +8 bp *bamD* → / → *raiA*, +GC *gltP* → / ← *yjcO*	4	0.01	0.5	0.5	R. Mutzel lab (Blattner et al., 1997)
S1	Δ26,518 bp in *lpxL*	4	0.02	0.5	0.5	Nordholt et al.^19^
S2	Δ6,314 bp in *lpxL*	8	0.02	0.5	0.5	
S3	*rssB, lpxM*	8	0.02	0.5	0.5	
S4	*rssB, lpxM*	8	0.02	0.5	0.5	
S5	*opgC /opgG, lpxM*	4	0.02	0.5	0.5	
S6	*opgH, lpxM*	4	0.02	0.5	0.5	
WT-mCherry	attTn7::*npII*-mCherry	n.d.	n.d.	n.d.	n.d.	this study
WT-YFP	attTn7::*npII*-YFP	n.d.	n.d.	n.d.	n.d.	this study
S4-mCherry	attTn7::*npII*-mCherry	n.d.	n.d.	n.d.	n.d.	this study
S4-YFP	attTn7::*npII*-YFP	n.d.	n.d.	n.d.	n.d.	this study
WT∆*ompF*	Δ216bp in *ompF*	n.d.	0.18	n.d.	n.d.	this study
S4∆*ompF*	Δ216bp in *ompF*	n.d.	0.16	n.d.	n.d.	this study
BW25113 (wildtype)	Δ*lacZ*, Δ*araBAD*, Δr*haBAD*, *rph, rrnB, hsd*,	n.d.	0.02	n.d.	n.d.	Keio collection (Baba et al., 2006^37^); MIC determination this study
JW1844 (Δ*lpxM*)	BW25113 *lpxM*::FRT-Kan-FRT	n.d.	0.02	n.d.	n.d.	Keio collection (Baba et al., 2006^37^); MIC determination this study

*AMP* Ampicillin, *CIP* Ciprofloxacin, *COL* Colistin, *GEN* Gentamicin, *n.d.* not determined.

### Tolerance to BAC limits evolvability of resistance to different antibiotics

**Fig. 2:**
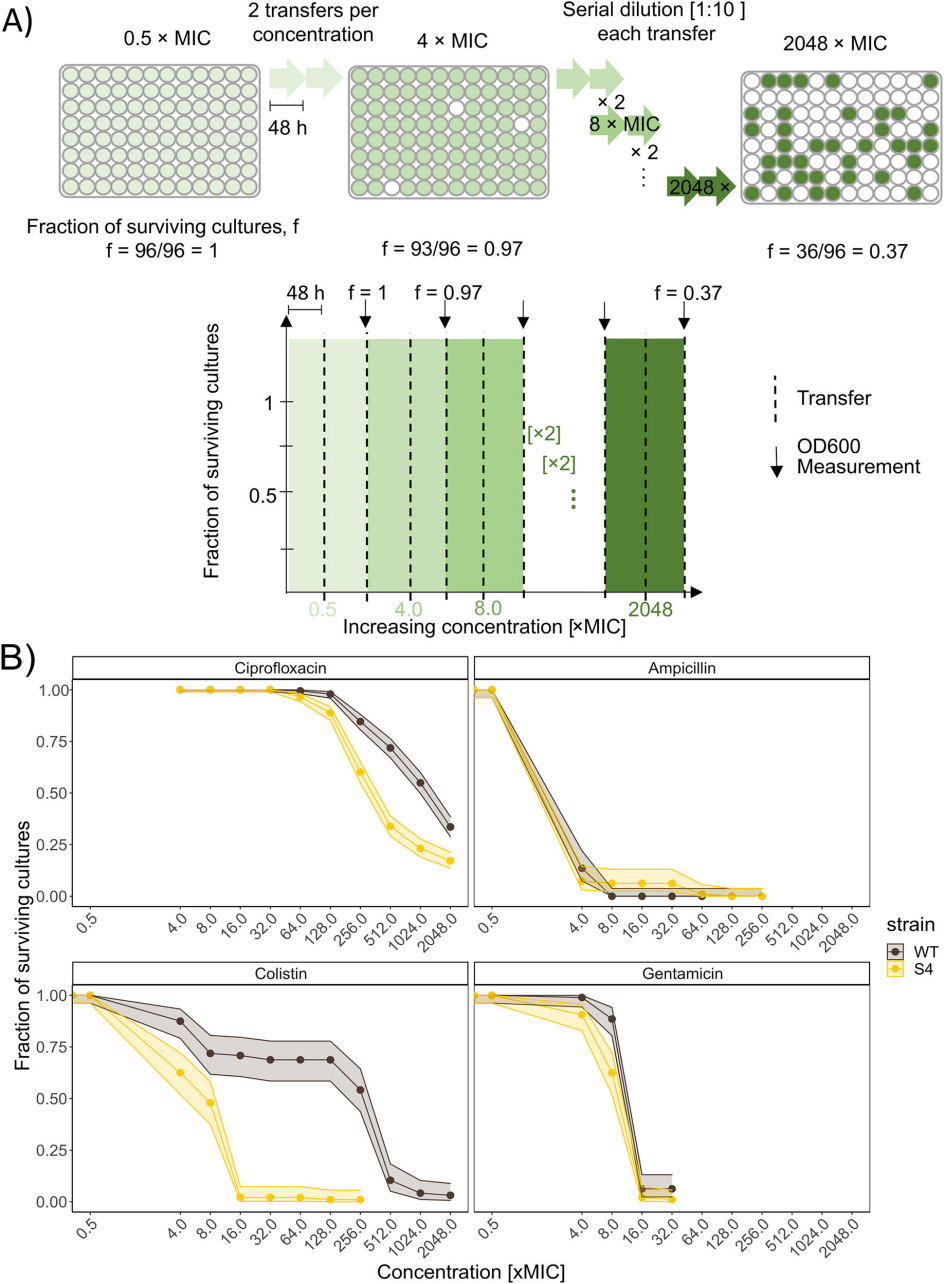
Benzalkonium chloride (BAC)-sensitive *E. coli* MG1655 (wildtype, WT) shows increased evolvability against antibiotics as compared to its BAC-tolerant S4 strain. **A** Scheme showcasing the design of the adaptive laboratory evolution experiment. The experiment was performed in 96-deep well plates. The cultures were initially incubated with a concentration of 0.5 × MIC, then the cultures were transferred to 4 × MIC, and thereafter the concentration was increased in twofold steps until 2048 × MIC. The cultures were transferred twice at each concentration and grown for 48 h after each transfer (dashed lines). OD_600_ was measured after the 2^nd^ transfer at any given concentration (arrows) and the fraction of growing (surviving) cultures f was determined. The cultures were diluted 1:10 at each transfer. **B** The data shows the fraction of replicate evolving populations of each strain that had detectable growth at increasing concentration of antibiotics during the adaptive laboratory evolution experiment. Adaption to different antibiotics is shown for a concentration range of 0.5 to 2048 × MIC of the WT. Error bands show the 95 % confidence interval calculated using the method from Clopper & Pearson^69^ for binomial data. *n* = 384 for ciprofloxacin and *n* = 96 for the other antibiotics. Clinical breakpoints relative to the MIC of the WT: CIP: 50 × MIC (MIC: 0.01 µg∙mL^−1^), AMP: 2 × MIC (MIC: 4 µg∙mL^−1^), GEN: 4 × MIC (MIC: 0.5 µg∙mL^−1^), and COL: 4 × MIC(MIC: 0.5 µg∙mL^-1^).

Biocides and antibiotics are applied in the same environment such as hospital or veterinary settings. Pathogens may evolve tolerance to disinfectants like BAC^19,23,34^. This adaptation poses a significant clinical challenge because these tolerant bacteria cannot be eradicated by routine disinfection, allowing them to spread and to cause infections in humans or animals. In turn, these infections need to be treated with antibiotics, whereby bacteria are again forced to adapt to high concentrations of antibiotics. This raises the question how the evolution of BAC tolerance affects the evolvability of such strains in response to clinically relevant concentrations of antibiotics. The evolvability of resistance to the antibiotics CIP, AMP, COL, and GEN for the BAC-tolerant strain S4 as compared to the WT was assessed by performing adaptive laboratory evolution experiments using 96 to 348 parallel serial dilutions every 48 h at increasing concentrations (from 0.5 × MIC to 2048 × MIC of the WT; Fig. 2), exceeding the clinical breakpoint of all the antibiotics used. This approach imposes a selective regime in which only resistant populations proliferate. Regular testing of growth at each concentration step yields information on the probability of resistance evolution (i.e. the evolvability) by calculating the fraction of growing wells relative to all inoculated wells and plotting this against the increasing antibiotic concentration (Fig. 2 B).

Surprisingly, despite the selective advantage of S4 in the presence of subinhibitory CIP (Fig. 1), the data shows that the WT has increased evolvability as compared to the BAC-tolerant strain S4 for CIP, COL, and modestly to GEN, while the evolvability to AMP was similar between both strains (Fig. 2). This highlights the differential consequences of BAC tolerance on selection and resistance evolution. Different BAC-tolerant strains were tested for their evolvability to CIP to investigate whether decreased evolvability to CIP was generally associated to BAC tolerance. Indeed, the data showed decreased evolvability of CIP resistance for all BAC-tolerant strains investigated (Supplementary Fig. S5). However, the ability to evolve resistance against CIP between the different BAC-tolerant strains varied (decreased evolvability tendency: S4 > S3 > S5 > S6/S1 > S2).

The difference between the strains in the response to CIP could be explained either by differences in mutation rates or by differences in accessible evolutionary paths between different genetic backgrounds of the BAC-tolerant strains as compared to the WT. Our data indicate that differences in mutations rates are not a likely explanation for the observed differences. Mutation rates µ were in a similar range between the BAC-tolerant strain S4 as compared to the WT (µ_WT_ = 8.83 ∙ 10^−12^ mutations/bp/generation; µ_S4_ = 1,75 ∙ 10^−11^ mutations/bp/generation, *p*-value = 0.168 likelihood-ratio test; Supplementary Fig. S7). Also, the BAC-tolerant strains S3 and S6, which have significantly higher mutation rates than the WT (µ_S3_ = 2.72*10^−11^ mutations/bp/generation and *p*-value = 0.014, likelihood-ratio test compared to the WT and µ_S6_ = 2.53*10^−11^ mutations/bp/generation and *p*-value = 0.016), were less likely to develop high CIP resistance. However, the mutation rates were measured in the absence of any antibiotics, while it has been shown that subinhibitory concentrations of CIP increase the mutation rate of *E. coli*^29^. Consequently, although we did not detect significant differences in basal mutation rates, we cannot exclude that mutation rates were differentially affected in the different strains during CIP exposure. However, the lack of detectable differences in basal mutation rates suggests that differences in mutation rates are not a likely explanation for differences in evolvability between strains. Thus, the difference in evolvability is more likely to be attributable to differences in genetic background of the BAC-tolerant strains.

All the BAC-tolerant strains have mutations in *lpxM* or *lpxL*, genes coding for acyl carrier proteins catalyzing the two last steps of Lipid A biosynthesis in the outer membrane^19,35,36^. LpxM catalyzes the addition of a myristoyl group to lipid A, a key component of the LPS matrix, which is crucial for maintaining outer membrane stability, regulating membrane permeability, and establishing negative cell surface charge. Additionally, the S3 and S4 BAC-tolerant strains had a loss of function mutation in the regulator of the general stress response (*rssB*), S1, S5 and S6 a mutation in the synthesis of osmoregulated periplasmic glucans (*opGH*) and S2 had a deletion of genes related to mobility and chemotaxis^19^(Table 1). No additional mutations that indicate adaptation to the general handling in the laboratory or the growth medium are present in these BAC-tolerant clones^19^.

Since all BAC-tolerant strains S1-S6 show different nonsynonymous mutations in *lpxM* or *lpxL* genes, we hypothesized that these genes are involved in decreased evolvability to CIP and that their absence should affect evolvability. To test this hypothesis, we compared CIP evolvability between a *lpxM* knockout strain JW1844 from the KEIO collection and its parental strain *E*. *coli* BW25113^37^. The results show that the disruption of the *lpxM* gene in JW1844 increased the evolvability to CIP as compared to the ancestor BW25113, especially at concentrations below 256 × MIC (Supplementary Fig. S6). This is in contrast to the apparent decrease in CIP evolvability in the BAC-tolerant strains S1-6 as compared to their ancestor strain MG1655. These results may be explained by the fact that the BAC-tolerant strains carry point mutations in *lpxM* or *lpxL*, which could alter protein function without fully inactivating it. In contrast, *lpxM* is disrupted in strain JW1844, potentially leading to more drastic phenotypic effects. Alternatively, the observed result may be specific to the BW25113 background, which exhibited lower evolvability of CIP resistance as compared to the ancestor strain (MG1655) of the BAC-tolerant strains (compare Fig. 2 and Supplementary Figs. S5 and S6). In addition, all BAC-tolerant strains carry additional mutations which might affect evolvability e.g. through epistatic interactions. This idea is supported by the observation that evolvability of CIP resistance differs between BAC-tolerant strains (Supplementary Fig. S5). Furthermore, this suggests that the changes in evolvability in the BAC-tolerant strains cannot be solely attributed to mutations in *lpxM/lpxL*.

In addition to the effects of CIP, we observed that the difference in evolvability between the BAC-tolerant and sensitive strains was even more pronounced for COL (Fig. 2, Supplementary Fig. S5). While CIP requires membrane permeability to reach its intracellular target, DNA gyrase, COL targets the outer membrane by interacting with LPS, a key component of which is lipid A^38^. Importantly, all BAC-tolerant strains harbor mutations in *lpxM* or *lpxL*, genes involved in the final steps of lipid A biosynthesis. The presence of these mutations could negatively impact the acquisition or fitness of COL and CIP resistance mutations, thereby reducing evolvability. While for COL this might be directly caused by incompatibility of mutations affecting LPS structure, the reason for changes in CIP evolvability is not as readily deduced. These observations highlight how adaptation to biocides can limit subsequent resistance evolution to antibiotics independent of the targeted cellular structures. Further studies dissecting these interactions and their molecular bases could provide deeper insight into the evolutionary trade-offs associated with biocide tolerance.

### Deletions in the *ompF* gene are significantly enriched in WT populations as compared to BAC-tolerant populations during adaptation to CIP

**Fig. 3:**
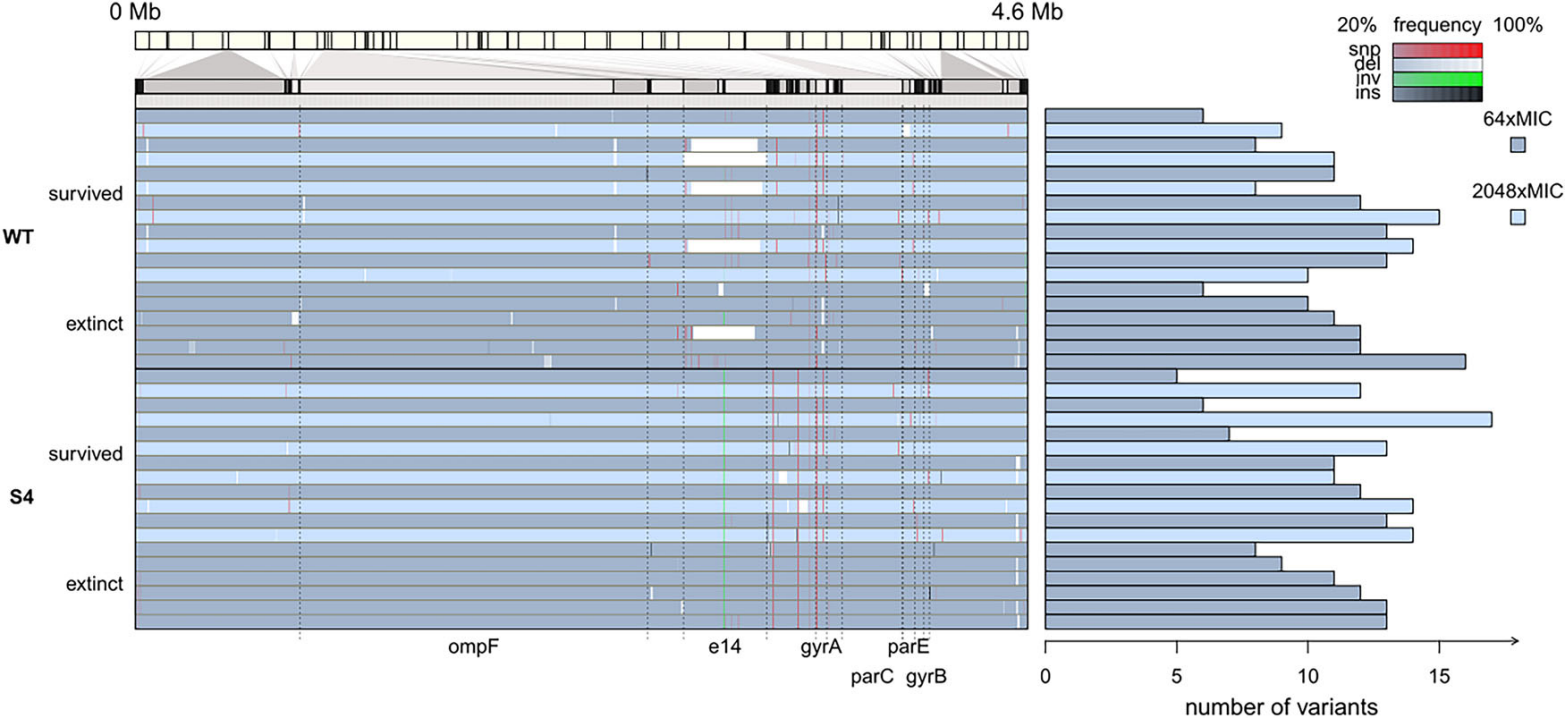
The *ompF* gene is preferentially mutated in the WT strain and not in the BAC-tolerant strain upon adaptive laboratory evolution to ciprofloxacin (CIP). Whole genome sequence analysis of *E. coli* MG1655 (WT) and its BAC-tolerant derivative S4 after experimental evolution in the presence of CIP. The bar at the top represents the genome of *E. coli* from 0 Mb to 4.6 Mb. Genomic loci with mutations are magnified in the panel below. Each of the 36 blue bars represent one replicate *E. coli* population from the evolution experiment. Dark blue bars show populations from the 64 × MIC concentration step of the experiment. The populations that adapted (‘survived’) to the highest concentration (2048 × MIC) of the experiment are represented by light blue bars. A dark blue bar (64 × MIC) followed by a light blue bar (2048 × MIC) represents sequences from a single evolved population at the two different concentrations. For both, the WT and S4, 6 populations that survived the entire evolution experiment (i.e. they adapted to 2048 × MIC) are shown. In addition, the 6 dark blue bars at the bottom of each strains’ segment (WT and S4) represent genomes of evolved populations at 64 × MIC that went extinct at concentration above 64 × MIC and below 2048 × MIC (‘extinct’). Red lines represent single nucleotide polymorphisms (snp), white lines represent deletions (del), green lines represent inversions (inv) and black lines represent insertions (ins). The intensity of the line shows the frequency of the specific mutation in the population. Names of key genes are indicated below the diagram. The number of variants in each population is shown on the right side of the figure.

Intrigued by the unexpected results demonstrating a selective advantage of the BAC-tolerant strain S4 in the presence of CIP and its concomitantly restricted evolvability of CIP resistance, we set out to uncover the genetic factors that restrict the evolutionary pathways towards CIP resistance in S4. To this end, we sequenced the whole genomes of 36 populations from the adaptive laboratory evolution experiments originating from WT and S4. We selected six populations originating from the WT as well as S4 that successfully adapted to the highest concentration of the experiment and six that went extinct before reaching the highest CIP concentration of the experiment. Of the populations that adapted to the highest concentration, we sequenced the DNA at two concentration-steps, 64 × MIC and endpoint of 2048 × MIC. Of the populations that went extinct before the endpoint, we sequenced the DNA only from the 64 × MIC concentration step. We detected mutations in 915 unique loci, at 2067 positions that were present in the population with a frequency higher than 5 % (Supplementary data file 1). For the statistical analysis, we focus on the mutations that were fixed in 20 % of the population or higher in order to reduce the noise by the plethora of rare mutations. A total of 70 genes at 132 positions were mutated with a frequency in the population of 20 % or higher across all populations (Fig. 3 and Supplementary data file 1). All sequenced populations had at least one mutation in the *gyrA* gene with 100 % frequency, except for one population that had a mutation in *gyrB* instead of *gyrA* (Fig. 3). The genes *gyrA* and *gyrB* encode for the DNA gyrase subunits A and B, respectively, which is the primary target of fluoroquinolone antibiotics in *E. coli*^39^. In addition, we observed a ~ 15 kb deletion in the WT, but not in the BAC-tolerant strain. Instead, we observed an inversion in the same area in the BAC-tolerant strain (Fig. 3 and Supplementary data file 1). The deleted region is the locus of prophage e14 which includes multiple genes (Supplementary data file 1) and its excision from the *E. coli* genome is a known stress response^40^. Statistical comparisons were carried out between the BAC-tolerant strain and the WT strain within each of the three sampling groups: 64 × MIC-extinct, 64 × MIC-survived, and 2048 × MIC. Additionally, comparisons were performed between the S4 populations that survived until the end of the experiment at 64 × MIC compared to 2048 × MIC, and the same comparison was conducted for the corresponding WT populations. Each gene that was mutated in the different groups was tested to determine whether it was mutated more or less frequently in one group compared to the other.

The statistical testing identified only one gene (*ompF*, encoding for the outer membrane porin F) that was mutated with higher prevalence in populations originating from the sensitive WT strain as compared to populations originating from the BAC-tolerant strain S4 (Pearson’s chi-squared test, *p* = 0.0273, *n* = 6, adjusted for multiple comparisons with the Benjamini & Hochberg, 1995 method^41^). In the populations that survived until the end, *ompF* was mutated in 5 of the 6 WT populations sampled from the 64 × MIC step and in none of the S4 populations at the same step. For the same populations sampled at the 2048 × MIC step, all 6 WT populations had mutations in *ompF* while only one of the 6 S4 populations from the same condition had acquired a mutation in *ompF*, which amounts to a small sample-adjusted unconditional maximum likelihood estimate of the odds ratio of 15 with the 95 % confidence interval 1.6–1422.7, calculated using the R package epitools^42^.

The whole genome sequencing data indicates that *ompF* mutations shape adaptation to CIP and that differences in *ompF*-based adaptation leads to differences in evolvability at high CIP concentrations. All the mutations in *ompF* were either deletions or insertions of length not multiple of three, causing a frameshift and consequently most likely rendering the gene inactive. The absence of OmpF was shown to contribute partially to resistance against another fluoroquinolone, norfloxacin, and was associated with a lower accumulation of norfloxacin inside of *E. coli* cells^43^. Although the MIC increase to CIP conferred by a *ompF* knockout is relatively low^44^, it is known that low resistance mutations can have positive epistatic effects with the major fluoroquinolone resistance mutations^45^. The WT populations that survived up to 2048 × MIC displayed four different mutations in the *gyrA* gene at 64 × MIC (D87G, Δ3 bp nucleotides: 247–249, S83L and G81D), while the BAC-tolerant strain had only two mutations in *gyrA* (D87G and S83L). This difference was not statistically significant in our experiment, but it is an indication that the occurrence of the knockout in *ompF* provides access to evolutionary pathways that enable specific mutations in the *gyrA* gene that are not accessible without the *ompF* loss-of-function. It should be noted that the high number of mutated genes compared to the number of sequenced populations affects the statistical power of the analysis. Thus, it is possible that other genes are differently mutated in the two strains but were not detected by our analysis.

### *ompF* loss-of-function has epistatic interactions with the BAC tolerance mutations

We hypothesized that the loss-of-function mutations in *ompF* are not selected in S4 populations because of epistatic interactions with the existing BAC tolerance mutations present in the S4 strain. Epistasis is a phenomenon in which the effect of one mutation is dependent on the presence of other mutation(s)^46^. Epistasis is widespread in nature, and it can be an important driver of the direction of evolution^47^. The relevant effects in the context of the evolution of antimicrobial resistance are related to the fitness of the genotypes, which can be measured e.g. as differences in growth rates or differences in susceptibility to antimicrobials measured as differences in MICs. Epistasis can be negative, whereby the measured effect of two mutations on fitness is stronger than the combined effect as predicted from the measured effects in the single mutants. Negative epistasis was detected previously in the context of antibiotic resistance acquisition^30^. In addition, another form of epistasis is called ‘sign epistasis’, which can occur if the sign of the effect changes e.g. from beneficial in single mutants to deleterious in the double mutant.

**Fig. 4:**
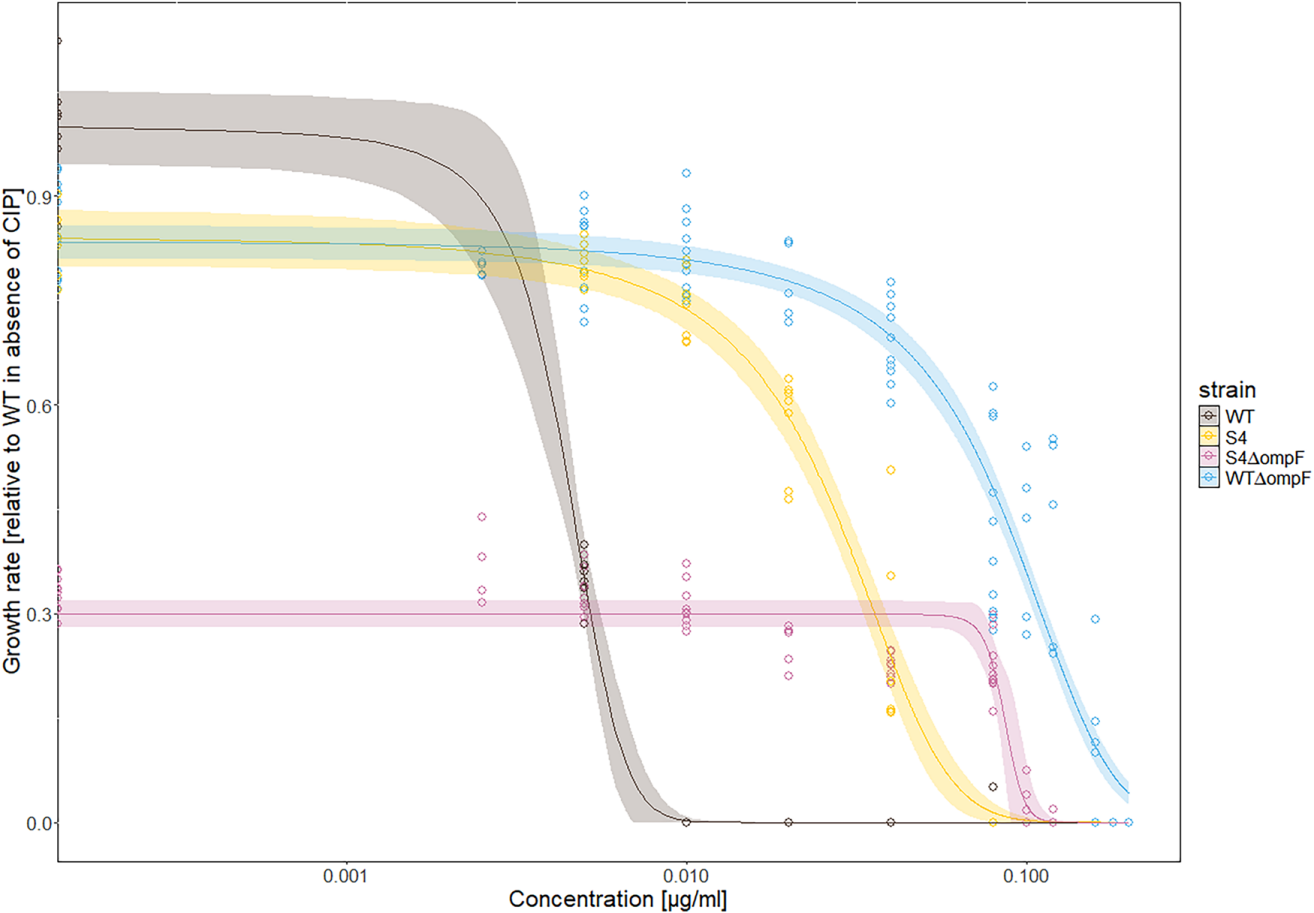
Dose-response curves to ciprofloxacin (CIP) of *E. coli* MG1655 (wildtype, WT), the BAC-tolerant S4 strain and their *ompF* knockouts. The y-axis shows the growth rate of the strains relative to the growth rate of the WT without CIP. The x-axis shows the CIP concentration on a logarithmic scale. The WT is represented in brown color, S4 in yellow, S4Δ*ompF* in pink and WTΔ*ompF* in blue. The lines show a logistic fit of the data, fitting 3 parameters: upper limit, the half-maximal inhibitory concentration (EC_50_) and slope. The lower limit was fixed to 0. The shaded area represents the 95 % confidence interval according to the fit. The model showed a strong predictive potential across all strains with *R*^2^ = 0.96 and *L*^2^ = 0.998, indicating that no linear corrections are needed to fulfill its predictive potential. The predictive potentials for each curve individually are as follows: *R*^2^ = 0.99 and *L*^2^ = 0.999 for WT, *R*^2^ = 0.96 and *L*^2^ = 0.997 for S4; *R*^2^ = 0.90 and *L*^2^ = 0.98 for S4∆*ompF*; *R*^2^ = 0.92 and *L*^2^ = 0.99 for WT∆*ompF*. The MIC values of the used strains as measured here are WT: 0.005–0.01 µg∙mL^−1^(median=0.0075), S4: 0.04–0.08 µg∙mL^−1^(median=0.06), WTΔ*ompF*: 0.16–0.18 µg∙mL^−1^(median=0.17), S4Δ*ompF:* 0.12–0.16 µg∙mL^−1^(median=0.14).

To test for epistatic interactions, we obtained knockouts of the *ompF* gene in the WT and the S4 BAC-tolerant backgrounds. We then measured and fitted dose-response curves for CIP of both strains. The results show negative epistasis of both mutations on the growth rates in the absence of CIP (i.e. overall fitness). Furthermore, the data shows sign epistasis relating to the effect on the MICs of CIP. Negative epistasis on growth rates was evident by observing a ~ 17 % reduction in growth rate for both S4 and WT-*∆ompF* relative to the WT. The expected fitness reduction of *∆ompF* in S4 would thus be ~34 %. However, the observed fitness reduction is ~70 %, demonstrating negative epistasis between the BAC tolerance mutations in S4 and a knock-out of *ompF* regarding growth rate. Sign epistasis on CIP susceptibility was evident by observing that increases in MICs as compared to the WT are 0.0525 µg∙mL^−1^ for S4 and 0.1625 µg∙mL^−1^ for *∆ompF* in the WT background, as calculated from the medians of the MIC estimation shown in Fig. 4. Thus, the expected combined increase in MIC would be 0.215 µg∙mL^−1^, while the observed MIC of *∆ompF* in the S4 background increases by 0.1325 µg∙mL^−1^. This demonstrates sign epistasis between the BAC tolerance mutations in S4 and the deletion of *ompF* because the presence of both mutations is deleterious (lower MIC) as compared to the beneficial effects (higher MICs) of the combined individual mutations. Specifically, the phenomenon would be classified as nonreciprocal sign epistasis because the effect on the double mutant fitness (S4*∆ompF* MIC = 0.14 µg∙mL^−1^) is lower than the effect in one of the single mutants (here WT*∆ompF* MIC = 0.17 µg∙mL^−1^). Taken together, the data show negative epistasis in terms of growth and nonreciprocal sign epistasis in terms of CIP susceptibility between BAC tolerance mutations in S4 and the deletion of *ompF*. These epistatic interactions are expected to limit the occurrence of mutations in *ompF* in the S4 background in the presence of CIP, because they will reduce the growth rate and increase the CIP susceptibility of emerging *ompF* mutants in S4 populations.

The literature confirms that *ompF* has role in CIP resistance in *E. coli* as well as other porins in different bacteria. For example, a reduced expression of *ompF* was observed in *E. coli* clinical isolates and from animals^44,48,49^, suggesting that it is a common pathway to high CIP resistance and not an artefact of our experimental design. In clinical isolates of *Klebsiella spp*., a reduced expression of major porins was also observed to be related to decreased CIP susceptibility^50^. *E. coli ompF* knockout mutants also showed slightly increased MICs to β-lactam antibiotics, tetracycline, chloramphenicol, and norfloxacin due to lower accumulation of the antibiotics in the cells^43,51^. Taken together, this shows that *ompF* mutations are expected to be beneficial during the evolution of CIP resistance and that limiting their occurrence in the BAC-tolerant strain can explain its reduced evolvability to CIP.

We note that solving the molecular mechanism of the epistatic interactions was beyond the scope of our work, but linking our results to the literature allows us to formulate hypotheses. All BAC-tolerant strains used for the evolution experiments have altered LPS. Interactions between the altered LPS and the absence of OmpF could explain the observed difference in antibiotic resistance evolvability between the BAC-tolerant strains and the BAC-sensitive WT. A large part of the surface of the outer membrane of *E. coli* is covered by β-barrel protein arrays next to patches of LPS^52^. OmpF is a β-barrel protein and one of the major outer membrane porins (OMP) in *E. coli*^52,53^. The absence of OmpF proteins could allow larger areas of the outer membrane to be covered by LPS and act as a barrier preventing the uptake of CIP^52^. The BAC-tolerant strain, having a mutation in LPS biosynthesis, possesses a different LPS composition. This altered composition in combination with the absence of OmpF could impair the function of the membrane, as the interactions between LPS and OMP are crucial for the integrity, stability, and assembly of the outer membrane^54^.

The second mutation that is present in the BAC-tolerant strain S4 is a loss-of-function in *rssB*, known to lead to a sustained activation of the general stress response via induction of RpoS^31,32^. RpoS not only activates a broad general stress response but also plays a crucial role in controlling mutagenic DNA repair pathways and thereby increasing mutation rates and potentially enhance evolvability^55,56^. However, elevated levels of RpoS and sustained general stress response activation do not necessarily translate into increased antibiotic resistance or evolvability because the regulatory network controlling stress-induced mutagenesis is highly complex. It involves at least 93 proteins acting upstream and within RpoS, RpoE, and related pathways, leading to outcomes that vary depending on the genetic background and environmental context^56^. Our data showed that there are no differences in mutation rates (Supplementary Fig. S7), but constrained evolvability towards CIP resistance (Fig. 2 and Supplementary Fig. S5). This suggests that additional molecular factors, including negative epistatic interactions—such as those involving *rssB*-controlled general stress response and *ompF* mutations—and other regulatory mechanisms likely limit evolvability or block key adaptive pathways.

Taken together, our data provide evidence for epistasis between mutations conferring BAC tolerance and mutations in *ompF*. The reduced evolvability connected with these epistatic interactions suggest that there is a common evolutionary pathway to high CIP resistance through the loss-of-function of *ompF*, which is blocked for the BAC-tolerant strain, because of these interactions, and thus the BAC-tolerant strain is less likely to evolve high CIP resistance.

In conclusion, biocides and antibiotics such as BAC, AMP, GEN, COL, and CIP are used jointly or are present in environments like hospitals, animal husbandry, and wastewater. Thus, BAC tolerance can evolve in these settings and the evolved BAC-tolerant bacteria can be exposed to antibiotics. Once BAC tolerance emerges, subinhibitory concentrations of CIP can select for BAC-tolerant *E. coli* strains over susceptible strains in those settings, and lead to the release of such strains into the environment. Contaminations with such strains could compromise the efficacy of BAC used for disinfection. Moreover, such BAC-tolerant *E. coli* strains may cause infections, which need to be treated with antibiotics. Our data shows that these BAC-tolerant strains are less likely to adapt to antibiotic concentrations that are relevant for such antibiotic treatments. One mechanistic explanation for this is through impaired access to a loss-of-function of *ompF* during evolution of CIP resistance. The deletion of *ompF* is not accessible in the BAC-tolerant strain because of epistatic interactions between the BAC tolerance mutations and the loss-of-function of *ompF*. In turn, the acquisition of the *ompF* deletion is a part of the evolutionary pathway to high CIP resistance. Taken together, our study highlights that the evolution of biocide tolerance will have intricate effects on the selection dynamics of such strains in the presence of antibiotics and their potential for de novo evolution of antibiotic resistance.

## Methods

### Bacterial strains, media, culture conditions and chemicals

All strains used in this study are listed in (Table 1). *E. coli* MG1655 (thereafter referred to as the ancestor or wildtype, WT) and its laboratory evolved BAC-tolerant mutants (S1 to S6) used in this study, were routinely grown on M9 minimal medium (M9: 42 mM Na_2_HPO_4_, 22 mM KH_2_PO_4_, 8.5 mM NaCl, 11.3 mM (NH_4_)_2_SO_4_, 1 mM MgSO_4_, 0.1 mM CaCl_2_, 0.2 mM uracil, 1 µg∙mL^−1^ thiamine, trace elements (25 µM FeCl_3_, 4.95 µM ZnCl_2_, 2.1 µM CoCl_2_, 2 µM Na_2_MoO_4_, 1.7 µM CaCl_2_, 2.5 µM CuCl_2_, 2 µM H_3_BO_3_) and 20 mM glucose) at 37 °C and 220 rpm. Where necessary, media were supplemented with ampicillin (AMP; Roth, K029.4), ciprofloxacin (CIP; Sigma, 17850-25G-F), gentamicin (GEN; Sigma, G1272-10mL), colistin sulfate (COL; Serva, 17420.02) or rifampicin (RIF; R3501-5G, Sigma-Aldrich). While introducing the fluorescent proteins to the *E. coli* strains, Luria-Bertani Lennox medium (LB medium) was used, which contained 10 g ∙ L^−1^ tryptone, 5 g ∙ L^−1^ yeast extract and 5 g ∙ L^−1^ NaCl. After the transformation of cells, super optimal broth (SOC medium) was used which contained 20 g ∙ L^−1^ tryptone, 5 g ∙ L^−1^ yeast extract, 0.5 g ∙ L^−1^ NaCl, 0.128 g ∙ L^−1^ KCL, 20 mM glucose and 40 mM MgSO_4_. The transposition of the transposon carrying the genes encoding the fluorescent proteins from the plasmids to the genomic DNA was induced by adding 0.1 % arabinose to LB medium. For serial dilutions, phosphate buffered saline (PBS) was used containing 10 mM (NH_4_)_2_SO_4_, 1.76 mM KH_2_PO_4_, 2.68 mM KCl and 137 mM NaCl. Plating and spotting were carried out on LB medium containing 1.5 % agar. Plates were incubated at 37 °C overnight unless stated otherwise.

### Introducing fluorescent proteins into *E. coli* WT and biocide-tolerant *E. coli* S4

To prepare strains for the competition assays, plasmids pMRE-103 (mCherry), and pMRE-105 (YFP)^57^ were transformed into NEB 5-alpha chemically competent *E. coli* cells (NEB C2987I) following the manufacturer’s protocol with slight modifications. Briefly, 3 µL of plasmid was added to 50 µL of thawed cells, mixed gently, and incubated on ice for 30 min. After a 30 s heat shock at 42 °C, cells were incubated in SOC medium at 30 °C for 90 min. Cultures were then plated on LB agar medium with 100 µg∙mL^−1^ AMP and incubated overnight at 30 °C. Plasmid isolation was performed using the Monarch Plasmid DNA Miniprep Kit according to the manufacturer’s instructions. The plasmid DNA was then quantified using a Nanodrop spectrophotometer (2000c, Peqlab). The plasmids were transformed into *E. coli* WT and BAC-tolerant *E. coli* strain S4 cells by electroporation. Cultures were grown to an OD_600_ of ~0.3–0.6 in LB medium at 37 °C with shaking. The cells were then washed with 10 % glycerol. The washing step was repeated for four times and the cells were resuspended in ~120 µL of 10 % glycerol for storage or immediate use. Electroporation was performed using a BioRad GenePulser with a 2.5 kV pulse. After adding 3 µL of plasmid DNA, the cells were transformed and recovered in SOC medium at 30 °C for 90 min. Transformed cells were plated on LB agar containing 100 µg∙mL^−1^ AMP and incubated overnight at 30 °C.

For genome integration of genes encoding fluorescent proteins, the protocol from Remus-Emsermann et al. 2016^57^ was followed. Briefly, plasmid-containing bacteria were grown in LB with AMP and 0.1 % arabinose to promote transposition. Colonies were screened for fluorescence with flow cytometry (Cytoflex S, Beckmann coulter) and confirmed by colony PCR using the attTn7 site-specific primers (5’GATGCTGGTGGCGAAGCTGT and 5’GATGACGGTTTGTCACATGGA)^58^. PCR was performed using OneTaq hot start master mix (M0484S, New England BioLabs) following the manufacturer’s protocol. The PCR cycle was set as following: initial 10 min step of 94 °C, 30 cycles of 30 s 94 °C, 30 s of 58 °C and 105 s of 68 °C. Next, a final extension step of 5 min 68 °C. The results were analyzed by 1 % agarose gel electrophoresis (Mupid-exU).

### Generation of Δ*ompF* knockout strains

To analyse the role of *ompF* towards the evolvability of CIP resistance, knockout mutants in the WT and BAC-tolerant strain S4 were created by using the CRISPR-Cas9 system as described in Jiang et al. 2025^59^ by the company SYNBIO Technologies. The removal of selection markers introduced during cloning was confirmed by plating on plates with the appropriate antibiotics. The deletion was confirmed by amplifying the flanking regions of the deleted target site using PCR and sequencing. The sequence information is provided in Supplementary text 1. Aligning the amplified sequence with the *ompF* gene shows a 216 bp deletion in the *ompF* gene (base pairs 596-812).

### Determination of minimum inhibitory concentration(MIC) and dose-response curves for antibiotics

The MICs for CIP were determined using a modified version of the broth microdilution method^60^. Briefly, an exponentially growing culture was diluted to ~10^7^ cfu ∙ mL^−1^ in a final volume of 200 μL M9 medium with increasing CIP concentrations. The following CIP concentration panels were used for different strains; JW1844 and BW25113: 0; 0.0025; 0.005; 0.01; 0.02; 0.04 µg∙mL^-1^, S4 and WT: 0; 0.005; 0.01; 0.02; 0.04; 0.08 µg∙mL^−1^, and S4Δ*ompF* and WTΔ*ompF:* 0; 0.0025; 0.005; 0.01; 0.02; 0.04; 0.08; 0.1; 0.12; 0.16; 0.18; 0.2 µg∙mL^−1^. Strains were incubated in 96-well microplates (polypropylene(PP), Greiner Bio One) at 37 °C for 24 h with shaking at 731 rpm in a BioTek Epoch 2 microplate reader. Optical densities were measured every five min and analyzed with the manufacturer’s software, Gen5 (version 3.09.). MIC values for the ancestor and strains S1 to S6 used in this study and additional antibiotics (CIP, AMP, COL, GEN) can be found in Table 1 and in Nordholt et al.^19^. Dose-response curves were constructed by calculating the growth rates from the growth curves using the Python-based algorithm described in Swain et al. 2016^61^. Dose response curves were fitted to the data with the drc package in R using a logistic formula and keeping the lower limit fixed to zero^62^. The goodness of fit of the data was evaluated by calculating the R-squared and L-squared statistics for non-linear models from the PAmeasures package^63^.

### Competition assay

Competitions between WT and biocide-tolerant strain S4 were conducted using WT-mCherry/S4-YFP and the reverse pair (WT-YFP/S4-mCherry) under four conditions per antibiotic in 96-well microtiter plates: M9 medium alone (control) and M9 medium with antibiotic (AMP: 0.5; 0.75; 0.85 µg∙mL^−1^, CIP: 0.0025; 0.005; 0.01 µg∙mL^−1^, COL: 0.2; 0.3; 0.4 µg∙mL^−1^, GEN: 0.05; 0.1; 0.2 µg∙mL^-1^). Nine overnight pre-cultures of each strain were grown in 200 µL M9 medium at 37 °C with shaking at 220 rpm (Kuhner X Climo-shaker ISF1-X). Aliquots of 1 µL of both strains were then added into fresh 200 µL M9 medium with the appropriate antibiotic, thereby establishing a 1:200 diluted 1:1 mixed culture from which a sample was immediately taken to determine timepoint *t* = 0. The cultures were incubated at 37 °C under shaking at 220 rpm for 24 h after which mixed cultures were then transferred into fresh medium with the appropriate concentration of antibiotics with a 1:100 dilution and grown again for 24 h. This cycle was repeated for three transfers in total. Samples were collected at the start of the experiment (*t* = 0, see above) and at the end of each growth cycle (i.e. *t* = 24; 48; and 72 h), diluted in 0.9 % NaCl solution, and analyzed via flow cytometry (Cytoflex S, Beckmann Coulter) to quantify fluorescent populations. All media used for flow cytometry were filtered through a 0.1 or 0.2 µm filter. The samples were excited at 561 nm and measured at 585/42 nm to detect fluorescence of mCherry and excited at 488 nm and measured at 525/40 nm to detect fluorescence of YFP. The logarithm of the ratio of BAC-tolerant cells divided by the number of ancestor cells were plotted versus the number of generations of the competition. For each replicate, the selection coefficient was determined from the slope of a linear regression fitted to the log-ratio across all timepoints. Non-linear regressions were performed using a logistic model in R (drc package)^62^ to fit the selection coefficient over the antibiotic concentration data from different competitions treatments. Using these models, the MSC was determined as the antibiotic concentration at which the competitive advantage of the biocide-tolerant strain became apparent (i.e. the concentration at which the selection coefficient is estimated to be 0).

### Adaptive laboratory evolution experiment

We developed a design to investigate the effect of biocide tolerance on the probability of resistance evolution to antibiotics in a high-throughput evolution experiment. The fundamental concept of this design is the stepwise increase of antibiotic concentrations above the MIC in a large number of parallel cultures (96 to 348) and then to measure the fraction of cultures that are able to grow. Thereby, the experiment assesses the probability of each strain to evolve high-level resistance (i.e. their evolvability). The evolution experiments were conducted with the WT and the BAC-tolerant strains (S1 to S6) to evolve resistance against the antibiotic CIP. To investigate CIP resistance evolution in *lpxM* knockout mutant, we used *E. coli* JW1844 ∆*lpxM* (JW1844) and its parental strain *E. coli* BW25113 (BW25113) from the Keio collection^37^. In addition, separate evolution experiments with WT and BAC-tolerant strain S4 were conducted with the antibiotics AMP, COL, and GEN.

Cultures were grown in 96-deep-well plates (1.2 mL, Ratio lab), serially transferred every 48 h at a 1:10 dilution in 700 µL final volume. The antibiotic concentration was initially set to 0.5 × MIC of the WT, increased to 4 × MIC and then increased twofold to a maximum of 2048 × MIC. At each concentration step, 70 µL samples were taken to assess growth photometrically at 600 nm using a microplate reader (Epoch2, Biotek). Cultures were considered growing if their OD_600_ exceeded the average of blanks (media without cells) plus three times the standard deviation. In rare cases, growth in wells at late transfers were preceded by the absence of growth of the same lineage in earlier transfers. In this case, the lineage was retrospectively considered to have grown in the early transfers without being detectable. Considering the relatively low dilutions (1:10) at each transfer, and the final cell density of our *E. coli* WT in M9 of ~10^9^ cells per 200 µL, statistically 9 transfers are necessary to exclude all the cells from a grown culture by dilution. It is possible that some cells acquire mutations that allow them to survive but only grow slowly and stay undetectable in the presence of the antibiotic. Subsequently such cells could acquire additional mutations to allow them to increase their growth rate, leading again to detectable growth. Stocks of cultures were prepared by adding glycerol to a final concentration of 15 % and storing them at -80 °C.

### Sequencing of evolved populations

To investigate the molecular mechanisms behind CIP resistance evolution, DNA was extracted from 36 populations originating from the evolution experiments, including 18 WT populations and 18 populations of the BAC-tolerant strain S4. These populations have either adapted to the highest CIP concentration or not. From six populations per strain that adapted to the highest concentration, DNA was extracted at both the 64 × MIC and endpoint (2048 × MIC) concentrations. In addition, DNA was extracted only at 64 × MIC for six populations per strain that did not reach the endpoint. DNA extraction was performed using the peqGOLD Bacterial DNA Kit (VWR) from 300 µL of thawed stock. One population required regrowth under the original experimental conditions (2048 × MIC CIP) to yield sufficient DNA. Illumina whole-genome sequencing was conducted by Eurofins, and mutations were identified using the breseq pipeline^64^ in polymorphism mode to extract mutations with frequencies between 0.05 and 1. The genome NC_000913.3 (NCBI RefSeq accession) was used for the variant calling. The sequence data has been deposited in NCBI under Bioproject number PRJNA1282584.

### Mutation rate determination

Mutation rates of the BAC-tolerant strains were determined using the Luria-Delbrück fluctuation assay^65^. The strains were inoculated from frozen glycerol stocks into LB medium and then grown at 37 °C with shaking at 220 rpm overnight. Overnight cultures were diluted to ~4000 cells mL^−1^ in fresh LB medium, and 54 independent cultures were inoculated with 200 µL of this dilution in 96-well plates. After growing the cultures overnight at 37 °C, 220 rpm, the entire volume of 48 cultures was plated undiluted on LB agar plates supplemented with RIF (100 µg∙mL^−1^) to determine the number of resistant mutants. Six cultures were diluted and plated on non-selective plates to estimate the total viable cell count per culture. After overnight incubation of the plates at 37 °C for 24 h, the number of colonies was counted. This setup allowed for the estimation of mutations rates with the maximum likelihood method, using the rSalvador package in R^66^. Statistical testing of differences between WT and BAC-tolerant strains S1 to S6 were performed with the likelihood-ratio test implemented in the rSalvador package at *p* < 0.05. RIF was used to determine mutation rates because the number of mutations that confer resistance to RIF are well described and thus allow accurate calculation of mutation rates^67^. The mutation rate per base pair was calculated by dividing the maximum likelihood estimation by 79 which is the number of mutations known to confer resistance to RIF^68^.

### Statistics

Statistical analysis was carried out in R version 3.6.1. The data from the competition experiments of each antibiotic-concentration combination were tested for normality with the Shapiro-Wilk test and optically by visualizing the QQ-plots. Not all data passed the test for normality, therefore the non-parametric One-sample, two-sided Wilcoxon test was carried out to test if the data are significantly different from 0. The *p*-values were corrected for multiple comparisons with the method from Benjamini & Hochberg^41^.

The data derived from the adaptive evolution experiments was used to calculate 95 % confidence intervals using the method from Clopper & Pearson^69^ for binomial data. Significance was assumed when the confidence intervals of the adaptive evolution experiments between the different strains were not overlapping.

Pearson’s Chi-squared test was used to test the difference in enrichment of mutations in genes in sequenced populations of the WT as compared to S4 at different stages of the adaptive evolution experiment. The *p*-values were corrected for multiple comparisons with the method from Benjamini & Hochberg^41^.

## Supplementary information

Supplementary Information

supplementary_data-revised.

## Data Availability

DNA sequences generated here can be found in NCBI under Bioproject number PRJNA1282584. All other data generated during this study are included in this article and its supplementary information files.
