# Supplementary material for: Consequences of benzalkonium chloride tolerance for selection dynamics and de novo resistance evolution driven by antibiotics
Source: NPJ Antimicrob Resist. 2026 Jan 8;4:2. doi: 10.1038/s44259-025-00170-8 (PMC12783665; doi:10.1038/s44259-025-00170-8)
Supplement: Supplementary file 1 — Supplementary Information [file 44259_2025_170_MOESM1_ESM.pdf]

Supplementary Information

Consequences of benzalkonium chloride tolerance for selection dynamics and *de novo* resistance evolution driven by antibiotics

Orestis Kanaris[a,#], Lydia-Yasmin Sobisch[a,#], Annett Gödt[a], Frank Schreiber[a,*], Niclas Nordholt[a]

[a]Division of Biodeterioration and Reference Organisms (4.1), Department of Materials and the Environment, Federal Institute for Materials Research and Testing (BAM), Berlin, Germany

[#]These authors contributed equally

[*]Address correspondence to Frank Schreiber and frank.schreiber@bam.de; Tel.: +49-30-8104-1414

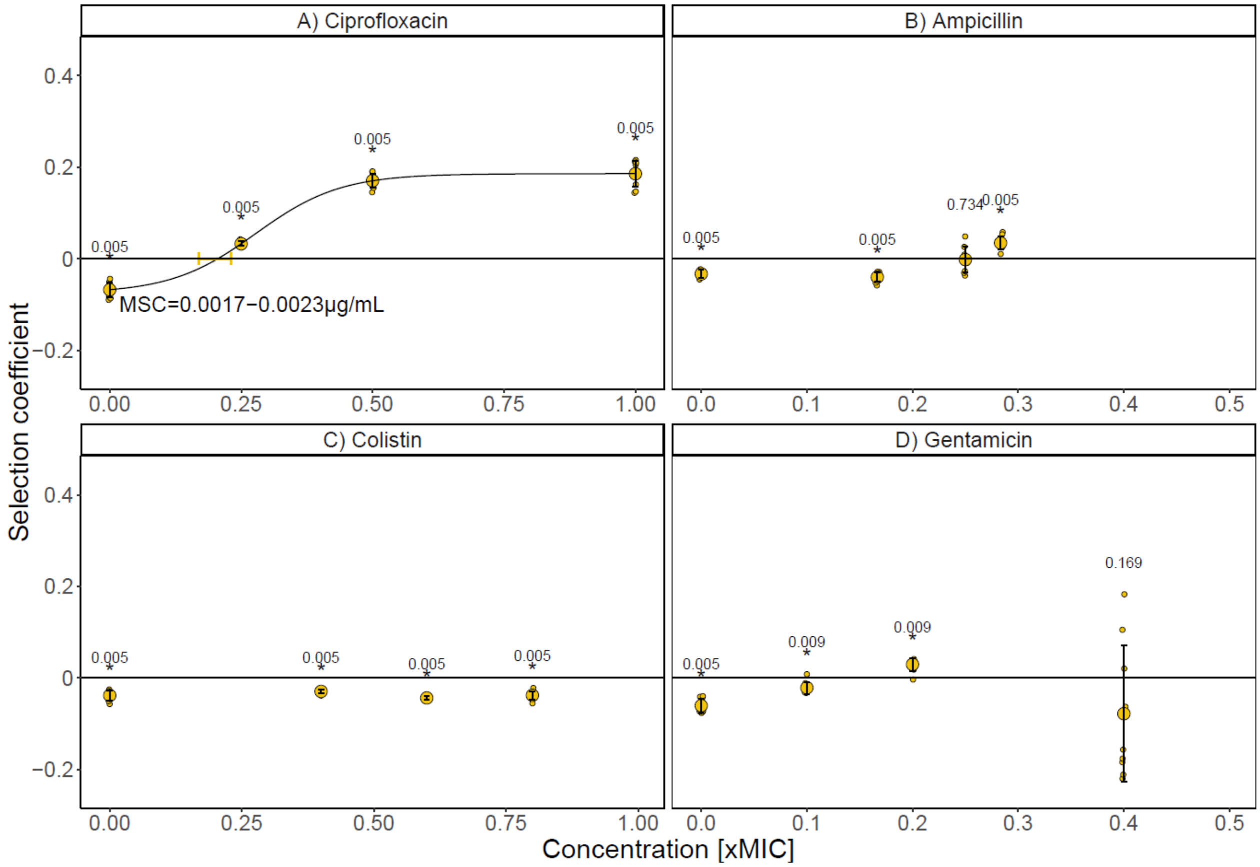

Supplementary Figure S1. The evolved, benzalkonium chloride (BAC)-tolerant strain S4 has a selective advantage over the *E. coli* MG1655 wildtype/parental strain (WT) under ciprofloxacin stress (A), but not in the presence of ampicillin (B), colistin (C) and gentamicin (D). Competitions between the WT tagged with YFP and the BAC-tolerant strain S4 tagged with mCherry were performed with 9 replicate lines under three different antibiotic concentrations (CIP: 0; 0.0025; 0.005; 0.01 µg·mL^{-1}, AMP: 0; 0.5; 0.75; 0.85 µg·mL^{-1}, COL: 0; 0.2; 0.3; 0.4 µg·mL^{-1}, GEN: 0; 0.05; 0.1; 0.2 µg·mL^{-1}). The panels display the selection coefficient of S4 against the WT as a function of the concentration represented as the fold difference to the minimum inhibitory concentration (MIC) of the WT (CIP: 0.01 µg·mL^{-1}; AMP: 4 µg·mL^{-1}; COL: 0.5 µg·mL^{-1}; GEN: 0.5 µg·mL^{-1}). The selection coefficient was calculated from the change of S4 relative to the WT over generations of competition (as calculated from data shown in Supplementary Figure S4). The regression of the experimental results (black line) in A was fitted with a logistic function including all nine competitions per concentration (small circles). The intercept with the x-axis represents the minimum selection concentration (MSC). Positive values on the y-axis indicate selection for S4 and negative values selection for the WT. The yellow, big circles show the mean of the nine individual replicates (small circles). The error bars show the standard deviations. Outcomes of competitions with reciprocal fluorescent tags are shown in Figure 1. One-sample Wilcoxon test was performed on the data of the 9 replicates to test for a significant deviation from 0 and the p-values are shown above each concentration. Stars indicate significance at p<0.05 corrected for multiple comparisons with the method from Benjamini & Hochberg[40].

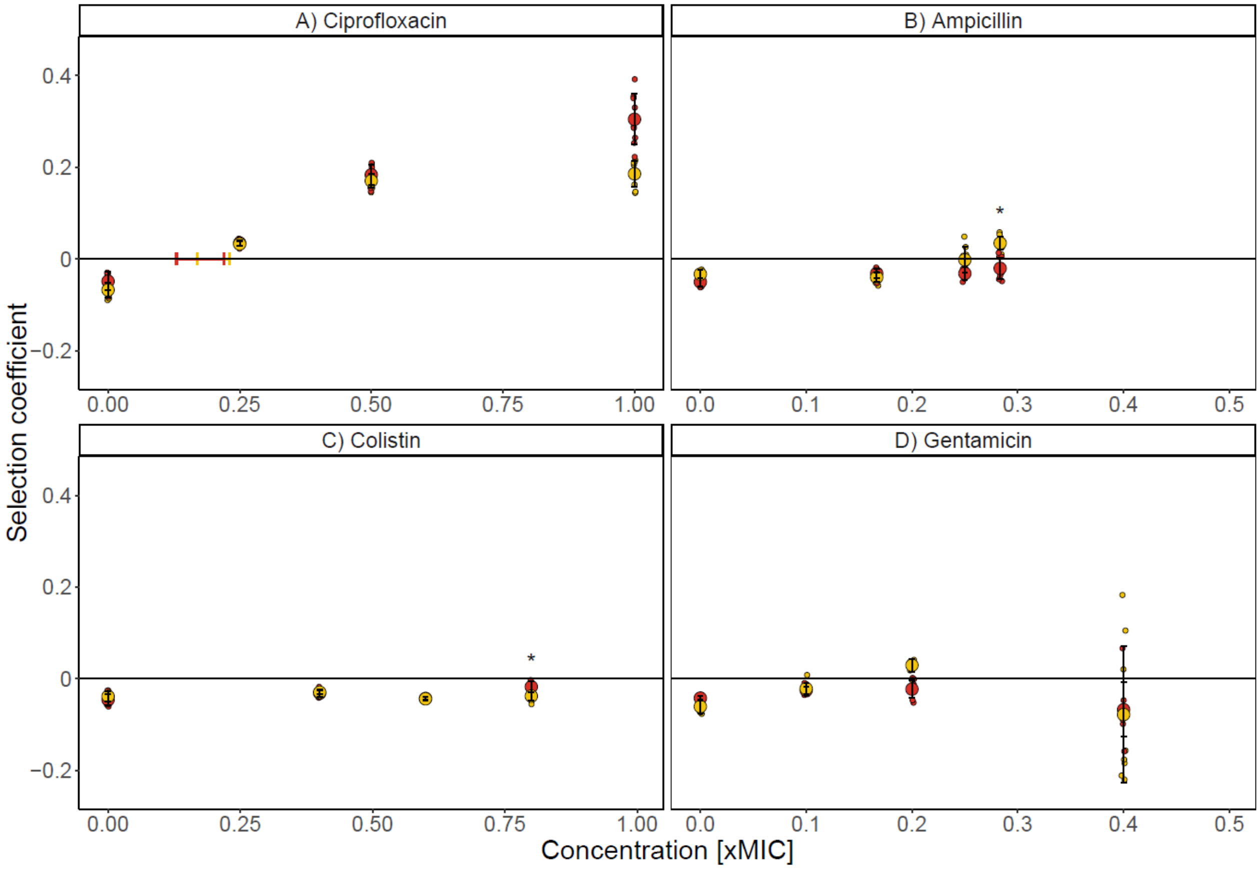

Supplementary Figure S2. The evolved, benzalkonium chloride (BAC)-tolerant strain S4 has a selective advantage over its ancestor (*E. coli* MG1655 wildtype, WT) in the presence of ciprofloxacin (CIP) stress (A), but not in the presence of ampicillin (B), colistin (C) and gentamicin (D). Competitions between the WT tagged with mCherry and the BAC-tolerant strain S4 tagged with YFP (red) and between the WT tagged with YFP and the BAC-tolerant strain S4 tagged with mCherry (yellow) were performed with 9 replicate lines per fluorescence combination under three different antibiotic concentrations (CIP: 0; 0.0025; 0.005; 0.01 µg·mL^{-1}, AMP: 0; 0.5; 0.75; 0.85 µg·mL^{-1}, COL: 0; 0.2; 0.3; 0.4 µg·mL^{-1}, GEN: 0; 0.05; 0.1; 0.2 µg·mL^{-1}). The panels display the selection coefficient of S4 against the WT as a function of the concentration represented as the fold-difference to the minimum inhibitory concentration (MIC) of the WT (CIP: 0.01 µg·mL^{-1}; AMP: 4 µg·mL^{-1}; COL: 0.5 µg·mL^{-1}; GEN: 0.5 µg·mL^{-1}). The selection coefficient was calculated from the change of S4 relative to the WT over generations of competition (as calculated from data shown in Supplementary Figures S3 and S4). Positive values on the y-axis indicate selection for S4 and negative values selection for the WT. The big circles show the mean of the individual replicates per fluorescence combination. The error bars show the standard deviation. Scheirer–Ray–Hare (from the R Package 'ecompanion') test was performed to compare the two fluorescence combinations per antibiotic and concentration. Post-hoc analysis was done using a Dunn test (from the R package 'FSA') for the antibiotics where significant differences were observed from the Scheirer–Ray–Hare test. The p-values from the Dunn test were corrected for multiple comparison with the method from Benjamini & Hochberg[42]. Stars indicate significance at $p<0.05$.

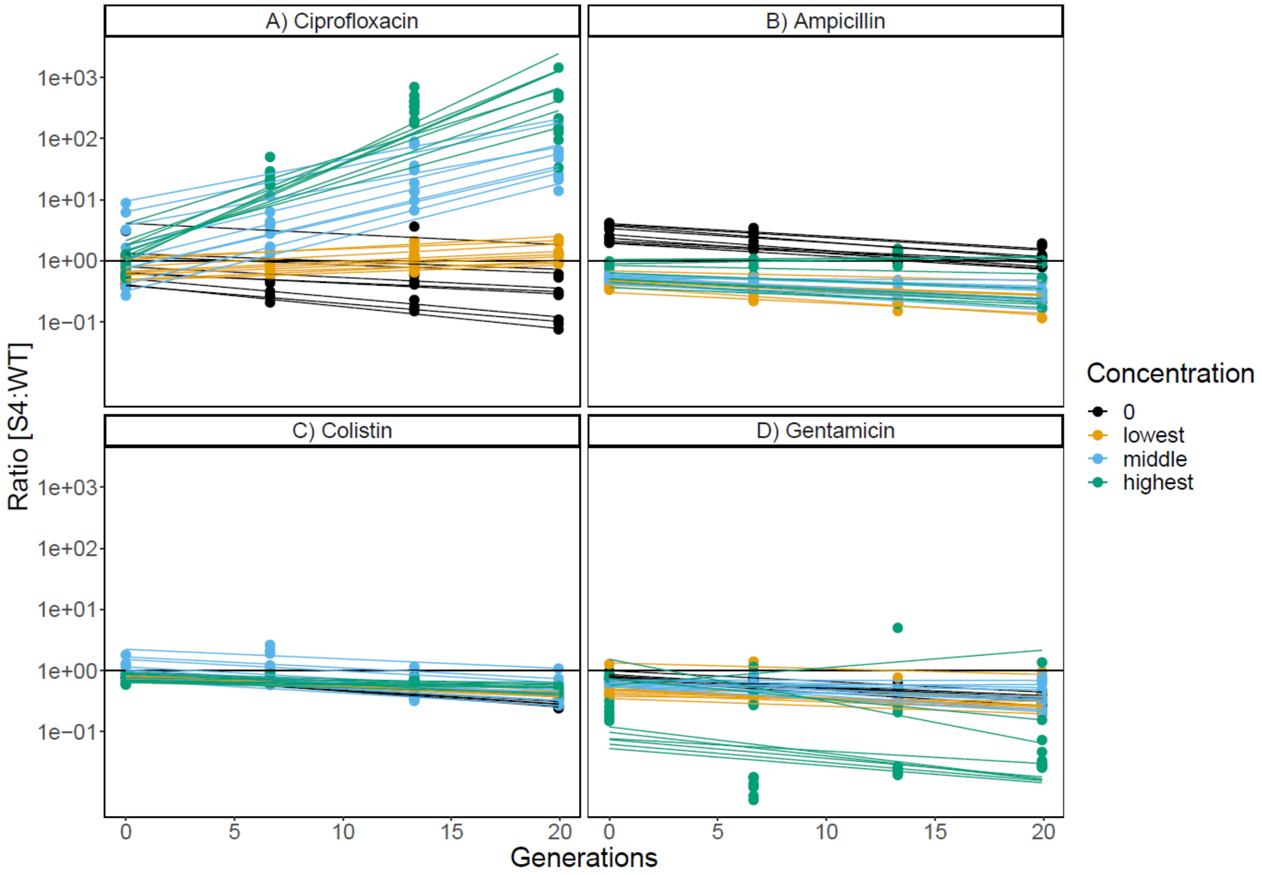

Supplementary Figure S3. Population dynamics of the competition experiment in the presence of four different antibiotics between the wildtype (WT) strain expressing mCherry and the BAC-tolerant strain S4 expressing YFP. Each panel shows the ratio of S4 divided by the WT plotted against the generations of the competition experiment. The Y axis is logarithmic. Lines represent linear regressions fitted for each replicate of the experiment (n=9). The colors represent the different antibiotic concentrations. In black, the results in the absence of antibiotics are shown, in yellow, the lowest antibiotic concentration, in blue, the middle concentration used and in green the highest. The antibiotic concentrations used in the competition experiments are CIP: 0.0025; 0.005; 0.01 µg/mL, AMP: 0.5; 0.75; 0.85 µg/mL, COL: 0.2; 0.3; 0.4 µg/mL, GEN: 0.05; 0.1; 0.2 µg/mL. The slopes of the linear regressions are the selection coefficients shown in Figure 1.

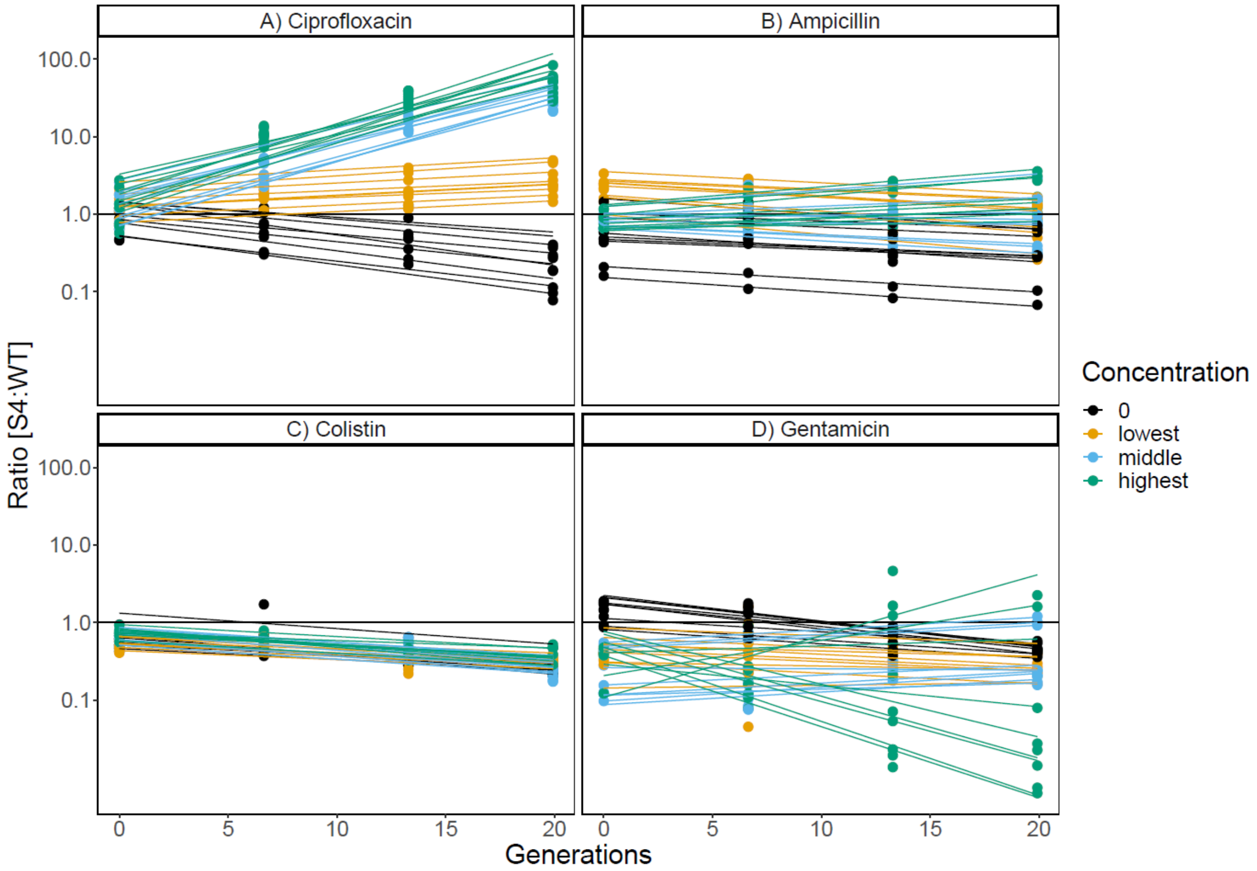

Supplementary Figure S4. Population dynamics of the competition experiment in the presence of four different antibiotics between the wildtype (WT) strain expressing YFP and the BAC-tolerant strain S4 expressing mCherry. Each panel shows the ratio of S4 divided by the WT plotted against the generations of the competition experiment. The Y axis is logarithmic. Lines represent linear regressions fitted for each replicate of the experiment (n=9). The colors represent the different antibiotic concentrations. In black, the results in the absence of antibiotics are shown, in yellow, the lowest antibiotic concentration, in blue, the middle concentration used and in green the highest. The antibiotic concentrations used in the competition experiments are CIP: 0.0025; 0.005; 0.01 µg/mL, AMP: 0.5; 0.75; 0.85 µg/mL, COL: 0.2; 0.3; 0.4 µg/mL, GEN: 0.05; 0.1; 0.2 µg/mL. The slopes of the linear regressions are the selection coefficients shown in Supplementary Figure S1.

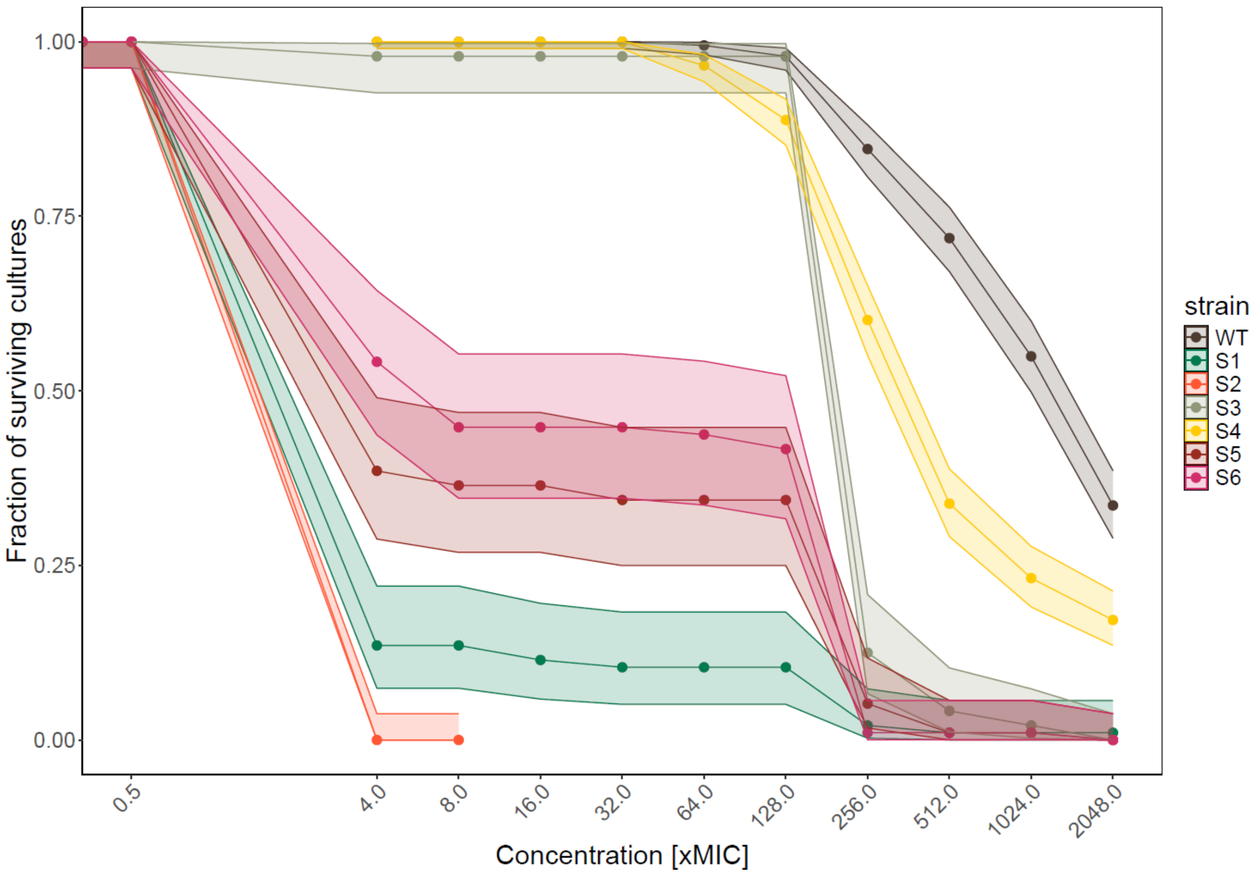

Supplementary Figure S5. Benzalkonium chloride (BAC) sensitive *E. coli* MG1655 (wildtype, WT) shows increased evolvability against ciprofloxacin as compared to different laboratory-evolved BAC-tolerant strains. The data shows the fraction of replicate evolving populations of each strain that had detectable growth at increasing concentration of ciprofloxacin in a serial transfer adaptive laboratory evolution experiment. Adaption to ciprofloxacin is shown for a concentration range of 0.5 to 2048 x MIC of the WT. The strains S1-S6 are BAC-tolerant. Error bands show the 95 % confidence interval calculated using the method from Clopper & Pearson[41] for binomial data. n=384 for WT and S4 and n=96 for all other strains.

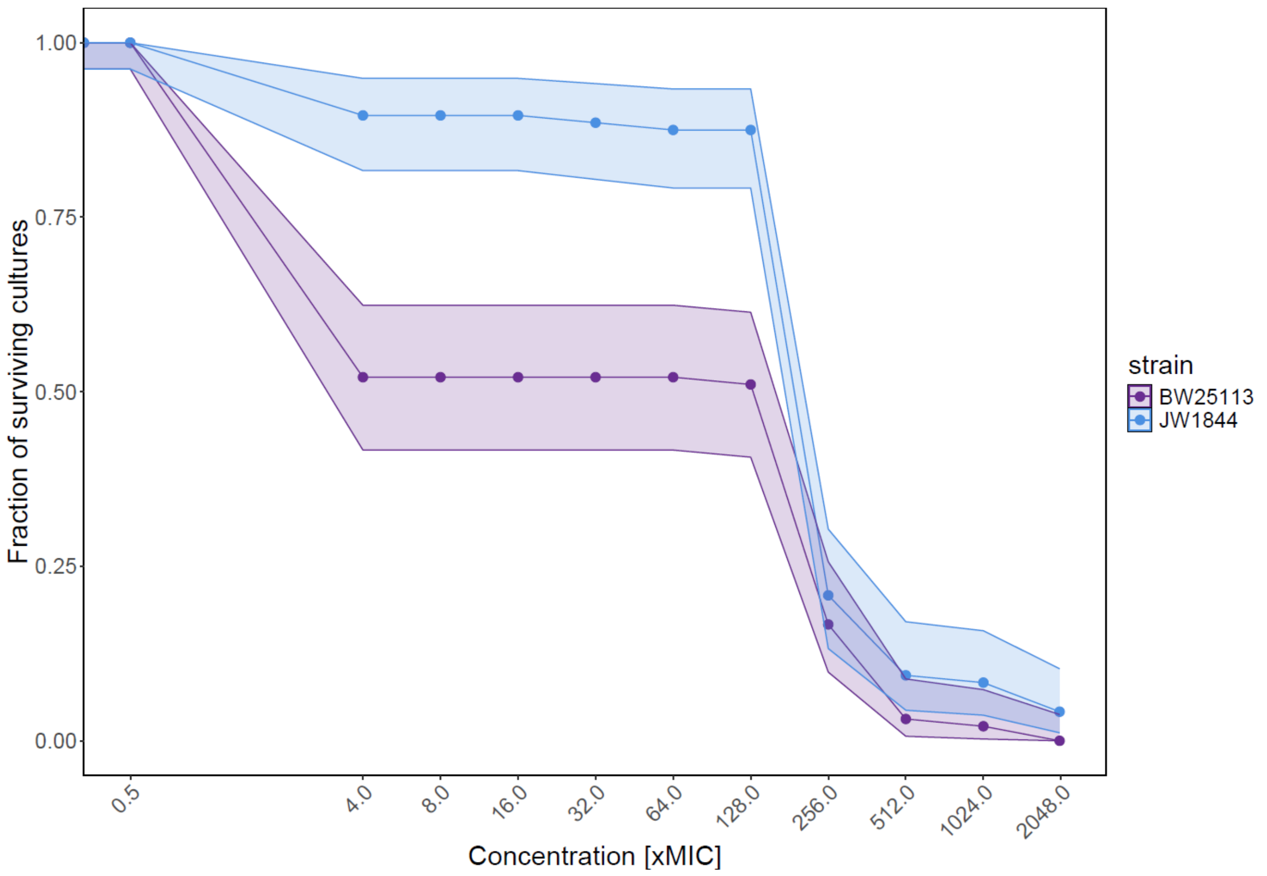

Supplementary Figure S6. *lpxM* knockout strain JW1844 shows higher evolvability of ciprofloxacin resistance than its parental strain BW25113. The data shows the fraction of replicate evolving populations of each strain that had detectable growth at increasing concentration of ciprofloxacin in a serial transfer adaptive laboratory evolution experiment. Adaption to ciprofloxacin is shown for a concentration range of 0.5 to 2048 x MIC of the WT. The strain JW1844 has a knockout in *lpxM* and BW25113 is its parental strain. Error bands show the 95 % confidence interval calculated using the method from Clopper & Pearson[41] for binomial data. n=96.

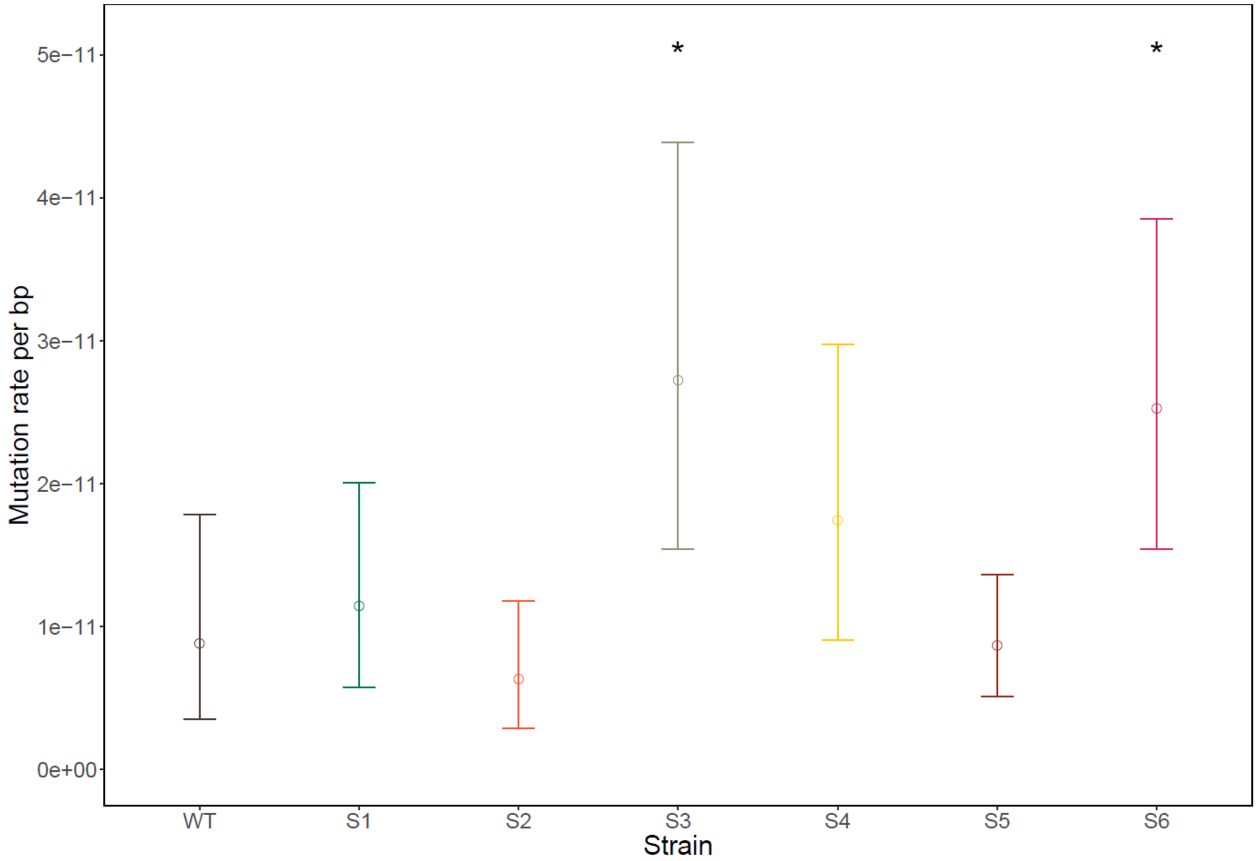

Supplementary Figure S7. Mutation rates of the WT and the BAC-tolerant strains. The data show the per base pair rates for each strain estimated by maximum likelihood method. The error bars represent the 95 % confidence intervals. The stars designate the strains with a statistically significant difference in mutation rate compared to the WT ($p < 0.05$) as tested with Likelihood ratio test. Stars indicate significant differences (S3 $p = 0.014$ and S6 $p = 0.016$).

Supplementary text 1

The sequence flanking the deletion in the *ompF* gene in the strain WTΔ*ompF* is:

____ACCGATTCCTTTCTTCCGTCTTTCGCTTTAGATTTGGTGTAAGCGATGGACGGACGCAGACCGAAATCGAACTG
GTATTGCGCAACTAACAGAACGTCTTGCGTTTTGTTGGCGAAGTTAACCGCCAACACCGTCGCCGTTAGAACGGCGT
GCAGTGTCACGCTCGTTTTTACCCAGGTACTGAACAGCGAAGTTCAGGCCATCAACCAGACCAAAGAAGTTGGAGTT
ACGATAGGTAGCAACGCCGCCAACACGACCAACGAAGGGGTCATCGC____

The highlighted base indicates the region of the deletion of a 216 bp segment from the original *ompF* gene.

The sequence flanking the deletion in the *ompF* gene in the strain S4ΔompF is:

____CCAGTATAACTTCCCGATTCCTCATTACGTCTCCCGCTATCAGGATTTGGTGTAAGCGATGGACGGACGCAGACC
GAAATCGAACTGGTATTGCGCAACTAACAGAACGTCTTGCGTTTTGTTGGCGAAGTTAACCGCCAACACCGTCGCCG
TTAGAACGGCGTGCAGTGTCACGCTCGTTTTTACCCAGGTACTGAACAGCGAAGTTCAGGCCATCAACCAGACCAAA
GAAGTTGGAGTTACGATAGGTAGCAACGCCGCCAACACGACCAACGAGGAGATCATCGC___

The highlighted base indicates the region of the deletion of a 216 bp segment from the original *ompF* gene.

Supplementary data file 1: Mutations of sequenced populations. Includes all mutations that were detected with a frequency 5% or higher in the sequenced populations from the evolution experiment.

Supplementary data file 2: Percentage of YFP and mCherry emitting populations. Includes the data that resulted from flow cytometry for the evaluation of the competition experiments.

Supplementary data file 3: Optical densities of evolving populations. Includes the optical density measurements of all the parallel populations during the evolution experiments.

Supplementary data file 4: Mutations of sequenced populations. Includes all mutations that were detected with a frequency 5% or higher in the sequenced populations from the evolution experiment. It contains the same information as Supplementary data file 1, but additionally, it includes the position of the mutations in the reference genome and the annotations of mutated genes, which is necessary to recreate Figure 3.

Supplementary data file 5: Gowth rates in the presence of CIP. Includes the growth rate measurements of the strains WT, S4, WTΔompF and S4ΔompF in the presence of different concentrations of CIP.

Supplementary data file 6: Mutation rate measurement. Includes the colony counts during the mutation rate measurement.
